# Meeting report of the seventh annual Tri-Service Microbiome Consortium Symposium

**DOI:** 10.1186/s12919-024-00307-z

**Published:** 2024-11-07

**Authors:** Zachary S. Liechty, Richard T. Agans, Robyn A. Barbato, Sophie M. Colston, Monica R. Christian, Rasha Hammamieh, Melissa R. Kardish, J. Philip Karl, Dagmar H. Leary, Camilla A. Mauzy, Ida Pantoja-Feliciano de Goodfellow, Kenneth Racicot, Jason W. Soares, Blake W. Stamps, Charles R. Sweet, Sara M. Tuck, Jordan A. Whitman, Michael S. Goodson

**Affiliations:** 1 711th, Human Performance Wing, Air Force Research Laboratory, Wright‐Patterson AFB, Dayton, OH USA; 2 United States Army ERDC Cold Regions Research and Engineering Laboratory, Hanover, NH USA; 3 United States Naval Research Laboratory, Washington D.C., USA; 4 Medical Readiness Systems Biology, Walter Reed Army Institute of Research, Silver Spring, MD USA; 5 Military Nutrition Division, United States Army Research Institute of Environmental Medicine, Natick, MA USA; 6 Soldier Effectiveness Directorate, United States Army Combat Capabilities Development Command Soldier Center, Natick, MA USA; 7 Unites States Naval Academy, Annapolis, MD USA

**Keywords:** Human Microbiome, Environmental Microbiome, Microbiome Engineering, Model Microbiome Systems, Military Microbiome

## Abstract

The Tri-Service Microbiome Consortium (TSMC) was founded to enhance collaboration, coordination, and communication of microbiome research among DoD organizations and to facilitate resource, material and information sharing among consortium members, which includes collaborators in academia and industry. The 2023 annual symposium was a hybrid meeting held in Washington DC on 26–27 September 2023 concurrent with the virtual attendance, with oral and poster presentations and discussions centered on microbiome-related topics within five broad thematic areas: 1) Environmental Microbiome Characterization; 2) Microbiome Analysis; 3) Human Microbiome Characterization; 4) Microbiome Engineering; and 5) In Vitro and In Vivo Microbiome Models. Collectively, the symposium provided an update on the scope of current DoD and DoD-affiliated microbiome research efforts and fostered collaborative opportunities. This report summarizes the presentations and outcomes of the 7th annual TSMC symposium.

**Supplementary Information:**

The online version contains supplementary material available at 10.1186/s12919-024-00307-z.

## Introduction

The Tri-Service Microbiome Consortium (TSMC) was chartered in 2016 to enhance collaboration, coordination, and communication of microbiome research among Department of Defense (DoD) organizations and to facilitate resource, material and information sharing among consortium members. Towards those goals, and to discuss applications and implications of DoD-associated microbiome research, the TSMC hosts an annual symposium that includes subject matter experts from federal/state agencies, DoD-affiliates, academic institutions, and industry [1–6]. The 2023 annual symposium was a hybrid meeting with the in-person option held in Washington, DC on 26–28 September 2023 concurrent with the virtual attendance. There were 222 registered attendees (70% DoD, 10% non-DoD government, 10% academia, 8% industry, and 2% foreign military), and over the two-day symposium 31 speakers presented on microbiome-related topics within five broad thematic areas: 1) Environmental Microbiome Characterization; 2) Microbiome Analysis; 3) Human Microbiome Characterization; 4) Microbiome Engineering; and 5) In Vitro and In Vivo Microbiome Models (Additional file 1). Fifty-two percent of the speakers were female and 16% of the speakers were from underrepresented minorities. Twenty speakers were from DoD laboratories (Additional file 1), five speakers were from non-DoD government laboratories, three speakers were from academic institutions (one from the United States Air Force Academy and two from the United States Military Academy), two speakers were from foreign military research laboratories, and one speaker was from a non-profit industry partner. Twenty-four posters were also presented during the symposium (13 from DoD laboratories, six from non-DoD government laboratories, four from academia, and one from industry). This report details the symposium activities to facilitate communication of current research efforts and foster potential collaboration across scientific communities.

## Environmental microbiome characterization

The session on environmental microbiome characterization reviewed research relating to various biomes including wastewater, soil, terrestrial ice, and marine systems, beginning with a discussion on the patterns of wastewater microbiomes at four DoD installations. Wastewater-based Epidemiology (WWBE) is a set of techniques and approaches that allows for chemical and biological measurement of a combined, anonymized waste stream for legal, epidemiological, and investigational reasons [7]. Further, wastewater could be reclaimed for potable water reuse, using the microbiome as one of the indicators of system design success [8]. WWBE was highlighted at four installations profiled over 4 months. The samples were investigated by shotgun metagenomic sequencing of waste-sourced biomass, and assessed by taxonomic, read-based functional, and assembly-based functional analysis. The researchers found that the functional capabilities of the microbiome were more stable by location and time than the taxonomic structure. Certain functional capabilities had significant associations with specific wastewater sources; for example, dehalogenase activity was found specifically within an industrial waste stream. This suggests that wastewater may serve as a biomining source of novel capabilities and materials related to it. A presentation at the poster session further expanded on wastewater profiling and investigated the potential to quantify specific neurotransmitters from wastewater. The researchers were able to correlate specific events on a DoD installation with temporal spikes in serotonin.

Profiling soil microbiomes can lead to the discovery and development of biomarkers for various environmental contaminants [9]. The next presentation addressed the possibility of detecting nuclear activity through soil microbiome profiling. The researchers hypothesized that changes in the soil microbiome could be indicative of the presence and influence of nuclear activity chemical byproducts or ionizing radiation, which could in turn lead to the development of biomarkers for nuclear activity. The most widely used chemical reprocessing process in the nuclear fuel cycle is plutonium uranium reduction extraction (PUREX), which involves the uses of kerosene, tributyl phosphate, and nitric acid. Exposing soil communities to these chemicals produced significant changes to the abundances of phosphotriesterase genes and *Phyllobacterium spp.*. Additionally, exposure to ionizing radiation led to functional differences in soil communities in a dose-dependent manner, including changes in nitrogen cycling genes. Work is ongoing to look at other types of radiation source/activity and to see if these approaches are generalizable between locations and soil types, as well as to extend this research to transcriptomic approaches that may increase the sensitivity of the analyses. Microbial bioremediation of soil was also explored in the poster session. A poster presented research on the potential for microbial degradation of hexahydro-1,3,5-trinitro-1,3,5-triazine (RDX), a military explosive that has been known to contaminate the environment near ammunition production sites [10]. The researchers found that mixing an isolate capable of producing RDX-degrading enzymes with compost was able to degrade RDX contamination in soil, but more research is needed to understand the correct proportions to optimize degradation.

Climate change can lead to accelerated permafrost thaw, resulting in the activation of previously frozen microbes. The DoD has an interest in the Arctic regions and the impacts of climate change on the stability of the Arctic biome [11–13], which can be affected by a changing microbial activity. The effects of climate change on the terrestrial microbiome were investigated through a laboratory incubation study simulating the mixing of Arctic terrestrial ice communities following melt. Permafrost contains a variety of dynamic and heterogeneous distribution features, including frozen soil and ice wedges (i.e. soil-free ground ice formations) and thermokarst cave ice. Microbial communities [14, 15] and their metabolites [16] are heterogeneously distributed in permafrost and even less is known about the microbiomes within terrestrial ice. In a laboratory study, a variety of terrestrial ice types from the Cold Regions Research and Engineering Laboratory (CRREL) Permafrost Tunnel were melted and mixed to determine the microbial response to new interactions through mixing. There were lower microbial respiration rates (a measure of the overall activity of microbial metabolism in the community) in the mixed samples compared to the individual samples, suggesting that there was some disruption of the steady-state of the microbiomes associated with each ice when they were mixed. However, when nutrient-rich thermokarst cave ice was introduced to these mixes, microbial respiration increased. These results demonstrate that mixing of permafrost ice features upon melting does alter the geochemical dynamics of the community, and that these effects are sample source-dependent rather than simply being an effect of population dilution/quorum effects. Research utilizing the CRREL Permafrost Tunnel was also presented during the poster session, where efforts to mine isolates from the tunnel to discover novel antifreeze proteins was reported. Isolates from these permafrost locations did exhibit antifreeze characteristics and are being investigated further for specific antifreeze protein activity and other biotechnology applications.

Finally, a metagenomic analysis of biofouling communities on the surface of unmanned underwater vehicles (UUV) was discussed. These biofilms impact the efficiency, and capabilities maritime vessels. As such, research into the consortia of microbes that form these biofilms could improve mission efficiency, yet little is known about the formation and composition of these communities on state-of-the-art coatings. Preliminary analysis of UUV parts coated in silicon-based paints exposed to both summer and winter marine waters off Key West revealed possible trends in alpha diversity by testbed, which suggest spatially unique communities were present in the biofouled surface by location. It is yet unclear if these trends are repeatable on varied platforms, or generalizable to the hydrology or aquatic chemistry in different locations and conditions.

## Microbiome analysis

The Microbiome Analysis session covered a variety of topics from standardization of protocols and analysis methods, implementation and development of various analytical tools, and data presentation. A representative from the National Institute of Standards and Technology reviewed the institute’s efforts to validate methods and materials within the microbiome industry. These efforts include both internal and external inter-lab comparisons of methods and results, validation reference materials (i.e. reference DNA or mock communities), and development of internal standards for metagenome sequencing. Metagenome sequencing is inherently compositional [17, 18], which can lead to misinterpretation of results. To address this, the researchers added an internal standard of known quantity in the initial extraction, allowing for a reference point by which the rest of the community can be scaled. To validate the internal standard, the researchers extracted DNA from a dilution series of stool samples, and found that the internal standard allowed for the calculation of a scaled abundance that closely matched the dilution series. This method could more accurately allow researchers to understand absolute shifts in microbial communities compared to relative shifts.

Efforts to develop machine learning techniques to build predictive systems of disease state from the microbiome while incorporating host metadata were also highlighted. The researchers collated 63 datasets to train their model. Challenges associated with combining these datasets include accounting for differences in methodology between studies as well as variations in metadata collected between studies. Some of these challenges could be alleviated by imputation, either directly or using a K-nearest neighbors method. It was found that including metadata significantly increased the accuracy in determining disease states over using microbiome data alone. Features found to have higher importance in classifying samples based on disease state generally include the families *Rickettsiaceae*, *Legionellaceae* and *Campylobacteraceae*. Features with higher importance while classifying gastrointestinal (GI) diseases specifically include *Photobacterium phosphoreum*, *Helicobacter mustelae*, and *Chondromyces* spp*.*. Further refinement of these models will be important in their implementation in other disease states including sepsis, wound healing, viral infections, and treatment efficacy.

The session then transitioned to presentations utilizing various methods of microbiome analysis. The association of microbiomes with marine biofouling was explored. Copper is frequently applied as a coating to the hulls of ships to act as a biocide and reduce biofilm formation [19]. Copper leaches from these panels at 9.5 ug/cm2/day, allowing resistant bacteria to form biofilms over time. Observations from the field demonstrate an increased presence of microbial resistance to copper coatings, and understanding the mechanism behind this is necessary to maintain its efficacy as a biocide. Researchers submerged panels at various sites either coated or not coated with copper oxide (CuO) and isolated microbes from biofilms forming on the panels after four weeks. The microbial species were identified through 16S sequencing and grown in a gradient of CuO to test their resistance to copper. CuO-tolerant microbes identified included *Altererythrobacter luteolus*, *Erythrobacter aquimaris*, *Bacillus megaterium*, *Bacillus licheniformis*, *Priestia* sp., *Alkalihalobacillus algicola*, and *Microbulbifer* spp. The researchers plan to better characterize the copper-tolerant isolates through genomics and proteomics, and potentially identify the mechanism of copper resistance.

A meta-analysis of captive vs wild animals was then presented to better elucidate the effects of microbiome transmission between hosts. A number of studies comparing captive to wild or domesticated animals were included, a diversity of species ranging from mammals [20] to reptiles [21]. The meta-analysis identified a slight trend towards increased diversity in the microbiota of captive vertebrates, and a significant difference in beta diversity between wild and captive vertebrates generally. Future analysis on this dataset will include estimating the impact of microbial diversity on health outcomes, quantifying the contribution of inter-host microbial to host health, and applying the findings to human populations.

While many topics in this session discussed upstream analysis, a poster from the Indoor Environments Division of the Environmental Protection Agency focused on conveying analysis results in an informative and simple way to the general public. Plans were presented on a forthcoming website that takes a systems view by integrating knowledge about the built environment to aid the general population in understanding where contaminants are found, what normal levels of contaminants are, and how individuals can protect themselves.

## Human microbiome characterization

The session on human microbiome characterization covered various aspects of the interplay between human health, human performance, and the gut microbiome within military populations. Military exercises are often physically demanding leading to a significant energy deficit [22]. Severe energy deficits may contribute to increased gut permeability during military training [23] and, if sustained, can negatively impact organ and immune systems [24] and military performance [25]. understanding energy imbalance in military populations can improve force readiness. The first presentation of the session described a study inducing energy imbalance on a cohort of military participants to identify the effects on the gut microbiome. To induce an energy deficient state, the research participants expended five to six thousand kcals per day for 72 h, slept four hours per day, and ate either an energy balanced diet, or a diet resulting in an energy deficit of 45%. The energy deficit increased gut permeability, and while community diversity was largely unaffected, several taxonomic and functional changes were observed. These changes included increases in short chain fatty acid (SCFA) producers and decreases in methanogens, and alterations of microbial pathways that may impact intestinal permeability, cell stress, or nutrient insufficiency; demonstrating that failing to maintain energy balance can have negative impacts on GI health.

Dynamics between Travelers’ Diarrhea (TD) and the gut microbiome of deployed service members were also discussed. TD is a prevalent issue among deployed military personnel, with surveys revealing that 60% of personnel returning from deployment experienced diarrhea during deployment [26]. TD can lead to persistent GI issues [27] and increase the prevalence of antimicrobial resistance genes [28]. The dynamics between TD and the gut microbiome were investigated in a cohort of participants who deployed for two months to the Philippines for a training exercise. Participant gut microbiomes were profiled before and after deployment, and surveys were taken on GI issues experienced during deployment as well as their behaviors during deployment. The researchers found that the likelihood of experiencing TD while deployed was partially influenced by the behaviors of the participants, such as whether they consumed food and water from the local economy or military facilities. There were marked changes in the microbiome after deployment compared to the pre-deployment microbiome. Some taxonomic changes occurred more generally across participants, such as an increase in Firmicutes and a decrease in Bacteroidota, whereas other changes were dependent on whether the participant experienced TD or not. Some taxonomies were found to be more prevalent in participants who did or did not experience TD while deployed, including multiple *Lachnospiraceae* spp. Microbes more prevalent in the guts of participants who did not experience TD could potentially be investigated as TD-preventative probiotics in the future.

Travelers’ Diarrhea research was also addressed in an overview of the current and future human-associated microbiome research from the Defense Science and Technology Laboratory of the United Kingdom’s Ministry of Defense. Various programs were covered, including the Microbiome Adaptations to Soldier Training program, which seeks to study the intersection of stressors, performance, and the microbiome; TD and the potential to optimize the gut microbiome to protect against TD; and investigating the difference between different military populations, such as the Gurkha population in the United Kingdom and non-Gurkhas. Future projects include moving beyond the human gut to study skin microbiomes in the context of wound infections and investigating canine microbiomes in association with behavioral characteristics.

In addition to studies on active military, a longitudinal study profiling the microbiomes of veterans was presented. The United States-Veteran Microbiome Project collected 5,570 samples from over 400 participants in order to study various aspects of health among veterans [29]. This rich dataset allowed the researchers to refine methods by identifying bias caused by different sampling method and sequencing centers. Extensive metadata was collected which allowed the researchers to assess the effects of homelessness, marriage, race, age, diet, deployment period and length, education level, time in service, medicine, GI and other health conditions, ethnicity, lifestyle behaviors in their various analyses. Some of the factors found to significantly impact the microbiome of veterans include race, bipolar medicines, and diet. Most homeless veterans were found to have acute stress disorder and traumatic disorder, and understanding how various factors interact with the microbiome can lead to a whole-body health approach for veterans after military service.

Another longitudinal study on human microbiomes was presented during the poster session, which investigated the temporal shifts in the microbiomes of submariners during a seven-month deployment. The investigators found that microbiomes remain relatively stable, though there are various aspects of the microbiome that change over time. They also found that various aspects of the mood and food survey data significantly correlated with the microbiome, suggesting changes in mood might be due to changes in their microbial communities.

## Microbiome engineering

The Microbiome Engineering session covered various efforts to genetically alter or utilize microbes in a way relevant to military goals. Due to the variable nature of military operations, precision in controlling the activation of these microbes is critically important. Researchers investigated one potential mode of controlled activation by using electromagnetic fields (EMF) to cause the germination of sporulated *Bacillus subtilis*. Various electrical fields have previously been employed to affect spore germination of various microbes, leading to mixed results [30]. In this experiment, *B. subtilis* spores were systematically exposed to a range of EMF at varying conditions and the activation response was characterized. Spore germination was assayed by measuring dipicolinic acid (DPA), which is present in the spore core and is released during germination [31]. EMF exposure experiments started in the microwave EMF range; initial low frequency microwave EMF were not found to stimulate germination, though experiments utilizing a higher EMF are planned. Initial knockout targets involved with germination were identified for further experiments to better understand and utilize the germination process.

The session shifted focus to the use of microbes as military-relevant material. The next presentation focused on transmitting data across melanized fungal tissue. The conductive properties of melanin have been well established [32, 33], though the majority of research in this area is on direct current rather than alternating current. The researchers developed a mat of *Curvularia lunata* similar in properties to synthetic leather, which they termed self-assembled conglomerated ascomycotal biotissue (SCAB). Galvanic couplings were used to transmit electricity through the SCAB to characterize responses. Data was successfully transferred through the SCAB, though the error rate increased depending on the rate at which data was transferred. Future research will focus on modeling the SCAB to estimate signal transmissions and using a finite elemental approach to simplify the melanin biological structure.

Biomaterial production through engineered microbes were also presented during the poster session. One poster discussed the process of bio-cementation, a soil stabilization process that exploits microbial-produced calcium carbonate precipitation to cross-link soil particles. The microbe most commonly studied in relation to bio-cementation is *Sporosarcina pasteurii*, which has been shown to more efficiently produce calcium carbonate compared to other microbes [34]. This poster covered attempts to transform key genes from *S. pasteurii* into *B. subtilis* in order to increase the efficiency of other calcium carbonate-producing microbes.

The session concluded with talks focused on the prevention of *Pseudomonas aeruginosa* infection. *P. aeruginosa* is common in the environment, can cause serious infections in wounds, and its antibiotic-resistant nature makes it challenging to treat [35]. The first talk presented research attempting to prevent *P. aeruginosa* using an engineered non-pathogenic *E. coli* strain to produce nanobodies targeting various *P. aeruginosa* proteins. Two targets were selected: FliD, the flagellar cap protein responsible for slowing down motility, and PcrV, a component of the protein complex used to inject toxins into a host [36, 37]. The researchers have optimized biofilm formation and will begin testing the interactions of the engineered *E. coli* with *P. aeruginosa.*

The final talk took a different approach to *P. aeruginosa* mitigation with the use of viral bacteriophage therapeutics [38]. While the human gut microbiome has been well studied, the effects of bacteriophage on microbiome assembly and function are still not well known [39]. Three isolated phages were identified with the ability to lyse *P. aeruginosa* strain PA01 and prevent growth with an additive effect. The researchers plan to expand the scope of the study to identify phages capable of lysing *P. aeruginosa* strain P14, and have developed a system to examine the dynamics of these phages within a greater microbial community using *C. elegans* as a host. Future research will involve understanding how phage treatment affects the gut microbiome more broadly after treatment.

## In vitro and in vivo microbiome models

In vitro and in vivo microbiome models are rapidly emerging as critical microbiome research tools to inform human study design including dosing, duration, and efficacy of interventions as well as the examination of stressors unsuitable for direct human evaluation. These models can also be used to predict individual responses to stress and disease factors. To open this session, the development of an in vitro skin microbiome models termed SURFACE or Skin Microbiome Reconstruction For Assessment of Cutaneous Effects was discussed. Using SURFACE platforms, evaluations of skin microbiome-microbiome interactions, microbe health, and medical countermeasure screening can be conducted. While current tools lack tissue complexity [40], SURFACE integrates a complex tissue system with relevant skin microbiomes to present a model with more realistic structural and functional properties. Current SURFACE models utilize differentiated human keratinocytes in a complex layered tissue system stratified with cornification and has been used to evaluate over 90 + combinations and ratios of microbiome components, with a 5 microbial member community able to co-culture for up to 7 days. Data analytics evaluated using this cell system include transepithelial/transendothelial electrical resistance (TEER), microscopy, and RT-PCR as well as the examination of antimicrobial peptides and metabolites.

In vitro models of the human lower GI tract offer a platform to assess the influence of dietary inputs on gut microbiota. A DoD-unique in vitro fermentation model that simulates the human lower GI tract, termed GI-jA2COB (joint Army Automated Colon on a Bench), has been implemented to explore the influence of polyphenol and fiber blends to examine the additive beneficial effects on healthy gut microbiota states [41]. GI-jA2COB was seeded with fecal samples in independent colonic domain mode to mimic the conditions of the ascending colon (pH 5.5) and was grown in a nutrient rich, complex colonic medium. Changes in bacterial community dynamics and metabolic production were studied as a function of polyphenol and fiber blends, respectively, with the combined blends shown to have a broader positive impact on beneficial taxa growth. The addition of polyphenol:fiber blends increased production of SCFAs and antioxidant capacity, increased the growth of commensal taxa such as *Bifidobacterium, Dorea, Ruminococcus,* and *Lactobacillus* spp., and decreased proinflammatory compounds [41]. The polyphenol:fiber mixture provided an additive effect on positive gut states compared to the polyphenol or fiber blends independently. This work serves as a platform for new candidate intervention approaches toward dietary modulation of the gut microbiome to build resiliency to negative health and disease states. Various posters also discussed the use of in vitro fermentative models to explore dietary changes to the microbiome. One study compared a normal Western diet to an MRE (meal, ready-to-eat) diet, and found no significant changes in microbiome composition. Further supplementation to the MRE diet with resistant starch type 2 resulted in an increase in *Dorea* spp [42, 43].

The session transitioned to discussions on in vivo small rodent models for evaluation of gut microbiome, behavioral, and physiological responses to acute traumatic psychological stress. Subjects with Post-Traumatic Stress Disorder (PTSD) have been seen to have an inability to regulate gamma aminobutyric acid (GABA)-ergic pathways, leading to dysregulation of fear and memory [44]. Current PTSD therapeutics include GABA antagonists, which can mask symptoms, and antidepression and serotonin uptake inhibitors, but can have severe side effects. There are no established biomarkers for PTSD [45]. To address these issues, researchers examined the gut microbiome for potential use as an alternative therapeutic target as well as a potential clinical biomarker. A Sprague–Dawley rat model (both male and female) was employed with exposure to multiple stress factors, and fecal, tail blood and plasma, and multiple tissues were collected. Immune panels as well as genomics, transcriptomics, and proteomics for microbiome assessments were performed. Gender-specific stress responses were evident as were increases in heart lesions in both male and female rats post-stress. Interestingly, the impact of stress on the microbiome revealed increased alpha diversity in males only and changes in beta diversity were seen in males relative to females. Differences in absolute abundances spanning multiple phyla/class/genera including Gammaproteobacteria, which increased in stressed males, *Lactobacillus* spp., which decreased in only males, and *Bacteroides* spp., which increased in stressed females, and are potential targets to investigate the connection between the gut microbiome and PTSD.

The session closed with discussions on integration of machine learning with in vitro bioengineered platforms to predict military health outcomes associated with the human microbiome. Computational machine learning and microbiome analytics are being coupled to predict resiliency, with the first phase of the work centered on the wound microbiome and predicting wound healing as a function of microbial diversity. Disease prediction using a multi-task machine learning approach linked with prediction validation using engineered biomes is currently being investigated as well with preliminary in silico models designed using over 13,000 metagenomes from several disease types. Functionality of predictions are being examined using in vitro co-culture models including those with more robust 3D structures created through biological printing approaches to represent cellular features more accurately and reproducibly, such as brush border membrane on the villi, and integration with a representative gut microbial component. Coupling machine learning tools with the sophisticated in vitro co-culture models will enable the next generation of research that, after human validation, could serve to predict disease susceptibility and stress response before exposures. This could have significant impact in both civilian and military sectors.

## Conclusion

The 7th annual TSMC symposium highlighted the diversity of microbiome research occurring within or in association with the DoD and demonstrates the broad applicability of microbes and microbe-derived products in addressing DoD needs. Research covered investigations of microbes in various biomes ranging from the environment to military materials to the warfighter, and were explored across an array of models ranging from human studies to in vitro models to computational models. The Environmental Microbiome session highlighted how microbiome profiling can elucidate changing biomes, whether that be climate-change-induced melting in the arctic, soil contamination, or wastewater. These studies highlighted the dynamics of change in these environments and show how environmental monitoring can lead to effective and timely decision making. The Microbiome Analysis session demonstrated the importance of each step in managing a sample from initial processing through displaying results in an informative fashion. Presentations highlighted the rapid growth and changes occurring within bioinformatic processing, including the implementation of newer artificial intelligence and machine learning algorithms. As these algorithms generally become more popular, opportunities of implementing them in microbiome datasets will become more available, and the TSMC meetings provide an opportunity to present how these cutting-edge advancements can be utilized by the community. The Human Microbiome session demonstrated the unique DoD-relevant questions that can be addressed using military populations, while highlighting the potential importance of human microbiomes to military health and performance. The Microbiome Engineering session underscored the multiple ways in which the results found from environmental or human studies can be utilized to produce actionable interventions, emphasizing the synergy existing between the various aspects of research covered in each session of this meeting. Finally, the session on in vitro and in vivo models demonstrates how interventions can be mechanistically studied or fine-tuned, increasing confidence in specific interventions. While the various sessions highlighted multiple ways in which DoD microbiome research intersects, the conference also discussed how differences in sample processing, standards, and analysis can lead to challenges when comparing results or synthesizing multiple studies. Meetings of the TSMC provide an opportunity to standardize methods and measurements, similar to the standard measures taken by other government agencies such as the National Institute of Standards and Technology [46] and the Human Research Program within the National Aeronautics and Space Administration [47]. The meeting of the TSMC provides opportunities to collaborate, coordinate, communicate, and unite in the vision for microbiome research within the DoD going forward.

## Supplementary Information

Additional file 1. TSMC Annual 2023 Meeting Program. Meeting program of the 7th Annual TSMC Symposium containing the agenda and presentation abstracts.

## Abbreviations

CRREL: Cold Regions Research and Engineering Laboratory
CuO: Copper Oxide
DoD: Department of Defense
DPA: Dipicolinic Acid
EMF: Electromagnetic Fields
ERDC: Engineer Research and Development Center
GABA: Gamma Aminobutyric Acid
GI: Gastrointestinal
GI-jA2COB: GI-joint Army Automated Colon on a Bench
MRE: Meal, Ready-to-Eat
PTSD: Post-Traumatic Stress Disorder
PUREX: Plutonium Uranium Reduction Extraction
RDX: Hexahydro-1,3,5-trinitro-1,3,5-triazine
SCAB: Self-assembled Conglomerated Ascomycotal Biotissue
SCFA: Short-chain fatty acid
TD: Travelers’ Diarrhea
TEER: Transepithelial/Transendothelial Electrical Resistance
TSMC: Tri-Service Microbiome Consortium
UUV: Unmanned underwater vehicles
WWBE: Wastewater-Based Epidemiology
