# Supplementary material for: Meeting report of the seventh annual Tri-Service Microbiome Consortium Symposium
Source: BMC Proc. 2024 Nov 7;18(Suppl 20):25. doi: 10.1186/s12919-024-00307-z (PMC11542233; doi:10.1186/s12919-024-00307-z)
Supplement: Supplementary file 1 — Additional file 1. TSMC Annual 2023 Meeting Program. Meeting program of the 7th Annual TSMC Symposium containing the agenda and presentation abstracts. [file 12919_2024_307_MOESM1_ESM.pdf]

TSMC Annual 2023 Meeting Program

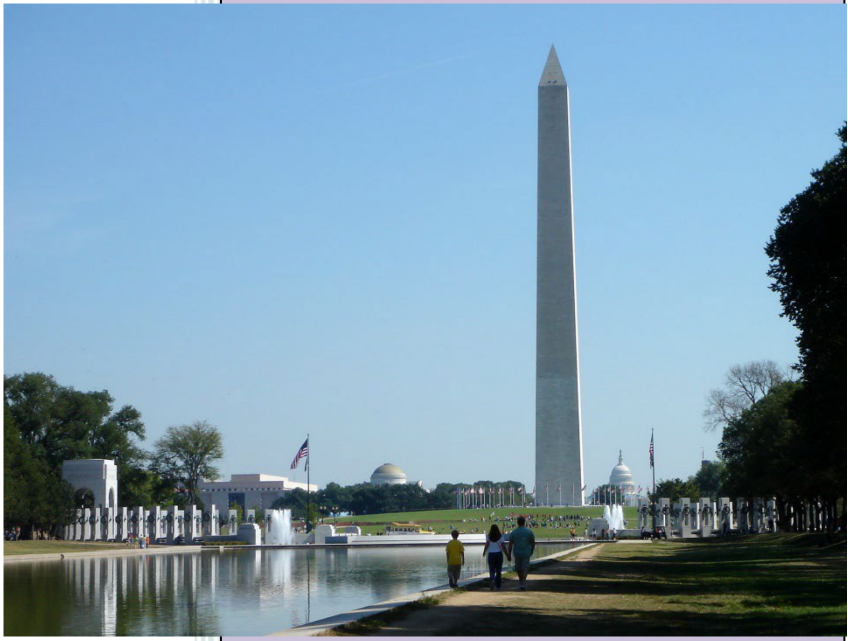

Hybrid Meeting | 25-27 Sep 2023

Sheraton Pentagon City,

Washington DC

TSMC

Tri-Service Microbiome Consortium

Welcome to the 7th Annual Tri-Service Microbiome Consortium Meeting

Meeting Purpose

On behalf of the Tri-Service Microbiome Consortium (TSMC), we welcome you to the Office of the Undersecretary of Defense (Research & Engineering), Biotechnology Community of Interest, 7th Annual TSMC Meeting: TSMC 2023! We are looking forward to two days of stimulating presentations and discussions from DoD researchers and our Government, Industry, and Academic partners.

The TSMC is a forum for DoD microbiome researchers to communicate ongoing research within the Army, Navy, and Air Force to identify research and capability gaps and coordinate research, while leveraging capabilities and resources. The annual TSMC meeting is designed to enable information sharing between DoD scientists and leaders in the field of microbiome science, thereby keeping DoD consortium members informed of the latest advances within the microbiome community and facilitating the development of new collaborative research opportunities. We publish the Annual Meeting Reports, so please check them out if you are interested in learning more about microbiome research within the DoD.

We encourage you all to take advantage of the interactive features of our hybrid event as much as possible to make TSMC 2023 as vibrant as usual. We also hope to see you at our no-host social on Tuesday evening!

We hope you find TSMC 2023 informative and useful.
Let the symbioses begin!

Michael Goodson | Chair, TSMC
711th Human Performance Wing
Air Force Research Laboratory,
Wright-Patterson Air Force Base, OH 45433

Dasha Leary | Vice-Chair, TSMC
Center for Biomolecular Science and Engineering
Naval Research Laboratory
Washington, DC 20375

Please check back often for updates

Event Resources

TSMC is about engaging and connecting with each other as well, and we have plenty of opportunities to join the conversation and network throughout this event. Join in during the live Q&As, use the chat to message everyone or direct message your colleagues, and continue the conversation on social media (#TSMC2023). To get the most out of TSMC 2023, make sure to explore your TSMC 2023 Attendee Guide and get familiar with the agenda.

This agenda is interactive! Click through the table of contents to get to that section. In the schedule, each of the speakers' names will take you to their abstract.

Past TSMC Meeting Resources

- **TSMC Annual Meeting Reports**

- 1st Annual Meeting
- 2nd Annual Meeting
- 3rd Annual Meeting
- 4th Annual Meeting
- 5th annual meeting:
    - Part 1
    - Part 2
- 6th Annual Meeting

- **Other TSMC Publications**

• Evaluation of Probiotics for Warfighter Health and Performance
[DOI: 10.3389/FNUT.2020.00070, AGANS, RICHARD T., ET AL. " FRONTIERS IN NUTRITION 7 (2020): 70]

• Gut Microbiota-Targeted Interventions for Reducing the Incidence, Duration and Severity of Respiratory Tract Infections in Healthy Non-Elderly Adults
[DOI:10.1093/MILMED/USAA261, KARL, J. PHILIP, MILITARY MEDICINE 186.3-4 (2021): E310-E318]

• Orally ingested probiotics, prebiotics, and synbiotics as countermeasures for respiratory tract infections in nonelderly adults: a systematic review and metanalysis
[DOI:10.1093/ADVANCES/NMAC086, COLEMAN, JULIE L., ET AL., ADVANCES IN NUTRITION 13.6 (2022): 2277-2295]

• Orally ingested probiotics, prebiotics, and synbiotics as countermeasures for gastrointestinal tract infections in nonelderly adults: a systematic review and metanalysis
[DOI:10.1016/J.ADVNUT.2023.02.002, FAGNANT, HEATHER S., ET AL., ADVANCES IN NUTRITION (2023)]

• Current advances in microbiome sciences within the US Department of Defense—part 1: microbiomes for human health and performance
[DOI: 10.1136/MILITARY-2022-002307, COLSTON SM, ET AL. BMJ MIL HEALTH PUBLISHED ONLINE FIRST: 15 JUNE 2023]

• Current advances in microbiome sciences within the US Department of Defense—part 2: microbiomes for human health and performance
[DOI: 10.1136/MILITARY-2022-002308, COLSTON SM, ET AL. BMJ MIL HEALTH PUBLISHED ONLINE FIRST: 18 JUNE 2023]

Tuesday, 26 Sep 2023 (all times US Eastern)

**0730-0830**	**Check-in/Login/Morning Social**
**0830-0840**	**Opening Remarks & TSMC overview**<br>Dr. Michael Goodson, Chair (AFRL)<br>Dr. Dasha Leary, Vice-Chair (NRL)
**0840-0855**	**OUSD(R&E) Biotechnology Overview**<br>Dr. Kate Sixt, Principal Director of Biotechnology, OUSD(R&E)
**0855-0910**	**Biotech Community of Interest Overview**<br>Dr. Peter Emanuel, Biotech CoI Chair (DEVCOM CBC)
**0910-0945**	**BioFutures 2050 Overview**<br>Dr. Diane DiEuliis, National Defense Univesity
	**Special Session – User Engagement #1**
0945-1020	User rep #1: Wg. Cdr. Jo Rimmer, UK RAF
**1020-1040**	**Morning Break**
**1040-1215**	**Technical Session #1: Environmental Microbiome Characterization**<br>Chairs: Dr. Charlie Sweet (USNA), Dr. Robyn Barbato (CRREL)
1040-1100	Dr. Blake Stamps (AFRL) 'Identification of Patterns in Temporally Resolved Wastewater Based Metagenomics'
1100-1120	Dr. Justin Podowski (Argonne Natl. Lab.) 'Biosignatures of Ionizing Radiation and Chemical Exposures in Soil Microbiomes'
1120-1140	Ms. Lindsay Gaimaro (CRREL) 'When Ancient Ice Microbiomes Meet: Measuring Microbial Activity from Experimental Mixing of Ground Ice Features from the Permafrost Tunnel in Fox, AK'
1140-1200	Dr. Melissa Kardish (NRL) 'Characterizing Unmanned Underwater Vehicle Biofouling Metagenomes to Combat Fouling Challenges'
1200-1215	Session Panel Q&A
**1215-1330**	**LUNCH**

**1330-1505**	**Technical Session #2: Microbiome Analysis**<br>Chairs: Dr. J. Philip Karl, (USARIEM), Dr. Sophie Colston (NRL)
1330-1350	Dr. Samuel Forry (NIST) 'DNA Internals Standards Improve Metagenomic Sequencing Analyses'
1350-1410	Mr. Morie Alpha (NSWC-IH) 'Tracking Inter-Host Microbial Transmission and its Impact on Microbial Diversity'
1410-1430	Dr. Hiranmayi Ranganathan (LLNL) 'Comprehensive Microbiome-Host Metadata Analysis for Disease State Classification'
1430-1450	Ms. Sara Tuck (NRL) 'Isolation and Identification of Copper-Tolerant Fouling Communities'
1450-1505	Session Panel Q&A
1505-1525	Break
**1525-1730**	**Poster Session**
1730-1900	'No host' social

Wednesday 27 Sep 2023 (all times US EST)

**0730-0830**	**Morning Social**
**0830-0835**	**Welcome (TSMC Chairs)**
**0835-0920**	**Special Session – ELSEI of Microbiome Research**
0835-0900	Dr. Robyn Barbato, US Army Corps of Engineers, Engineer Research & Development Center, Cold Regions Research & Engineering Laboratory
0900-0920	ELSEI Q&A
**0920-1030**	**Special Session – User Engagement #2**
0920-0940	User rep #3: SSgt. Arianna Baldwin, USAF
0940-1000	User rep #4: COL Robert Cybulski Jr., US Army
1000-1030	User Representative Panel Discussion
**1030-1050**	**BREAK**
**1050-1225**	**Technical Session #3: Human Microbiome Characterization**<br>Chairs: Dr. Rasha Hammamieh (WRAIR), Dr. Richard Agans (AFRL)
1050-1110	Dr. Cassandra Suther (USARIEM) 'Negative Energy Balance Alters Gastrointestinal Function and Gut Microbiota Composition and Function During Strenuous Physical Activity''
1110-1130	Dr. Zachary Liechty (AFRL) 'The Two-Way Interactions of Travelers' Diarrhea and the Gut Microbiome'
1130-1150	Dr. Lisa Brenner (Dept. Veteran Affairs) 'Six Years of Longitudinal Veteran Microbiome Sampling, Major Findings, and Lessons Learned: The United States-Veteran Microbiome Project (US-VMP) Study'
1150-1210	Dr. Emily Parish (UK DSTL) 'The Effect of Changes to the Gut Microbiome on Health and Performance''
**1210-1225**	Session Panel Q&A
**1225-1345**	**LUNCH**

**1345-1520**	**Technical Session #4: Microbiome Engineering**<br>Chairs: Dr. Blake Stamps (AFRL), Mr. Ken Racicot (DEVCOM SC)
1345-1405	Ms. Anne McCarthy (CERL) 'Electromagnetic Field as a Wake-up Signal for Sleeping Bacteria'
1405-1425	Mr. Caleb Shin (USAFA) 'Fighting Pseudomonas aeruginosa Wound Infections with an Engineered Skin Microbe'
1425-1445	Mr. Robert Jones (CRREL) 'Melanin in the Matrix: Transmission of Data Across Melanized Fungal Tissue'
1445-1505	Ms. Katie Herbert (West Point) 'The Effects of Bacteriophage Therapeutics for Pseudomonas aeruginosa Infection on the Microbiome in a Model Organism'
1505-1520	Session Panel Q&A
**1520-1540**	**BREAK**
**1540-1715**	**Technical Session #5: In vitro and in vivo microbiome models**<br>Chairs: Mr. Jason Soares, (DEVCOM SC), Dr. Camilla Mauzy (AFRL)
1540-1600	Dr. Else Vedula (Draper) 'Skin µbiome Reconstruction For Assessment of Cutaneous Effects (SURFACE)'
1600-1620	Dr. James DeMar (WRAIR) 'Evaluation of Gut-Microbiome, Behavioral, and Physiological Responses to Acute Traumatic Psychological Stress: A Rat Model Study'
1620-1640	Mr. Jordan Whitman (DEVCOM SC) 'In Vitro Fermentation shows Polyphenol and Fiber Blends have an Additive Beneficial Effect on Gut Microbiota States'
1640-1700	Dr. Nicholas Be (LLNL) 'Integrative Machine Learning and Bioengineered Platforms for Prediction of Military Health from the Human Microbiome'
1700-1715	Session Panel Q&A
**1715-1720**	**Closing Remarks (TSMC Chairs)**

ABSTRACTS LISTING

Technical Session #1: Environmental Microbiome Characterization

Identification of Patterns in Temporally Resolved Wastewater Based Metagenomics

Blake W. Stamps[1], Taylor Jones[2], James Christensen[2], Michael S. Goodson[2]
[1]Materials and Manufacturing Directorate, Air Force Research Laboratory, Wright-Patterson AFB, US
[2]711th Human Performance Wing, Air Force Research Laboratory, Wright-Patterson AFB, US

Wastewater is the amalgamation (and end-product) of many of our day-to-day activities including personal hygiene, cleaning, industrial activity, and more. These activities leave an indelible signal within the waste they generate. The signals in waste have recently been used to identify outbreak trends within SARS-CoV-2 utilizing wastewater-based epidemiology (WWBE) worldwide. WWBE proved invaluable during the pandemic to track when a city or location was likely to experience a surge in infections, often predating known clinical increases by 7 to 10 days. To this end, we collected wastewater from multiple locations over a 3 to 6 month period and tracked not only SARS-CoV-2 concentrations but also other respiratory viruses and most importantly, the total microbiome via deep metagenomic sequencing. We were able to identify the total bacterial, archaeal, micro-eukaryotic, and DNA based viral community of hundreds of wastewater samples during our sampling period as well as trends in the functional potential of these microbial communities over time. The use of environmental metagenomics will allow the expansion of WWBE to detect emergent pathogens but also allow for a greater understanding of the microbial ecology of our waste streams.

Biosignatures of Ionizing Radiation and Chemical Exposures in Soil Microbiomes

Justin C. Podowski, Sara Forrester, Derek R. McLain, Jennifer L. Steeb, Jamie C. Overbeek, Daniel S. Schabacker

Soil microbes have the potential to serve as important biosensors for nuclear activity. A better understanding of the way soil microbial communities respond to radioisotopes, ionizing radiation, or chemicals associated with nuclear reprocessing could allow us to leverage knowledge of those responses to determine if an environment has been exposed to any of those conditions. We have developed the infrastructure, workflow, and expertise to discover biosignatures in soil microbial communities to a wide array of conditions. Using amplicon and metagenomic sequencing of DNA, we demonstrate biosignatures specific to key chemicals in the PUREX process, as well as to ionizing radiation. Further, our methods can reliably determine limits of detection for and durability of these biosignatures. Beyond DNA, we use changes in soil microbe transcriptional profiles recorded in RNA to probe shorter term response of microbes present at the time of exposure to ionizing radiation. Use of RNA changes as a biosignature has greater methodological challenges but the potential to have much lower limit of detection than DNA. Altogether we present specific biosignatures in DNA and RNA as well as a platform to detect biosignatures of other conditions of interest.

When Ancient Ice Microbiomes Meet: Measuring Microbial Activity from Experimental Mixing of Ground Ice Features from the Permafrost Tunnel in Fox, AK

Lindsay W. Gaimaro[1], Logan M. Gonzalez[1], Robyn A. Barbato[1]
[1]ERDC Cold Regions Research and Engineering Laboratory, Hanover, NH

Massive ground ice features like ice wedges and thermokarst cave ice are abundant in the CRREL Permafrost

Research Tunnel in Fox, AK. These ice types form differently and often at different times which can influence the microbial and chemical composition in each type. Thermokarst cave ice usually forms laterally through the center of existing ice wedges: surface water or subsurface flow melts the center of the wedge without collapsing the structure. Water fills in this wedge cast and then freezes, creating two distinct ice types that will encounter each other if melting occurs. As the climate warms there is increasing chance of ground ice features melting across the sub arctic and arctic landscapes, which may lead to increased activity due to availability of previously inaccessible nutrients combined with an increase in metabolically active microbes. However, the microbial response to these melting events is not well studied, and it is unclear whether mixing of melt water from different ice types will tend to enhance or inhibit microbial activity. The mixing of melt water from these ice types in response to thaw will alter the chemical landscape, for example through the introduction of nutrients and changes in pH. Additionally, distinct microbial communities, which may have been separated for up to tens of thousands of years, will now interact. It is possible that this mixing will create community coalescence events that alter community assemblage dynamics and result in the mixed community resembling one of the starting communities more than the other, or perhaps an entirely separate community dynamic existing in an alternative stable state. To test this hypothesis, we created a laboratory incubation scheme in which melt water from three ice types (two ice wedges of different ages and one thermokarst cave ice) were mixed in different ratios with the idea that there is a threshold of introduction that needs to be met before we see differences in microbial activity and community dynamics. Once this threshold is crossed, we expect that the microbial respiration will be altered from the original activity and the communities will perhaps have reached an alternative stable state. After about 60 hours of incubation at 14 °C the respiration activity started to increase across all treatments. In general, the mixed treatments had lower respiration rates compared to each ice community by themselves, however, introduction of higher percentages of thermokarst cave ice to both ice wedge types increased respiration rates when compared to the lower percentages of thermokarst introduction. There was less of a clear pattern when mixing the two ice wedges together, suggesting that stochasticity might play a larger role in determining activity upon mixing. We expect to see patterns in the microbial community compositions that reflect these changes in microbial activity.

Technical Session #2: Microbiome Analysis

DNA Internals Standards Improve Metagenomic Sequencing Analyses

Samuel P. Forry, Stephanie L. Servetas, Jason G. Kralj, Jennifer N. Dootz

Background: Metagenomic sequencing (MGS) analyses have become a cornerstone of microbiome characterization. However, the resulting relative abundance data are inherently compositional in nature, such that the observed abundance of individual taxa are inherently correlated with one another and do not reliably represent actual genomic abundances. Herein, we propose the inclusion of DNA internal standards to correct for sample composition and improve measurement comparability between samples.
Methods: Pure DNA from 5 taxa was added to stool to serve as an internal standard for MGS analyses. These taxa (*L. pneumophila*, *A. baumannii*, *V. furnissii*, *N. meningitidis*, *A. hydrophila*) were chosen as they were not native to stool. Their DNA concentrations were varied across ~2 orders of magnitude. The DNA was mixed into stool samples from 5 donors ahead of DNA extraction and cleanup (Zymo Research Fecal DNA miniprep). Common MGS methods were employed for 16S amplicon (V34 library prep, genus assignment by dada2 using Silva v132 database) or shotgun (Nextera library prep, species assignment by Centrifuge using the Web Of Life database) sequencing. Each taxa's relative abundance (RelAbund) was normalized to the ratio of the RelAbund of one of the DNA internal standards and it's known starting concentration to produce a new metric, normalized abundance (NormAbund), which was compared quantitatively to RelAbund.
Results: The NormAbund metric was hypothesized to correlate with taxa actual abundances (as compared with RelAbund) and be independent of sample composition. To validate these hypotheses, we evaluated (i) spiked in taxa at constant actual abundance across 5 different stool compositions, and (ii) various taxa at systematically varied actual abundances and steady compositions. In each case, the NormAbund metric performed much better than RelAbund.

- <u>Constant actual abundances in varied sample compositions:</u> Observed RelAbunds for the internal standards were consistent for repeated analyses within a single stool composition (CVs ranged from 2% to 12%). However, between the 5 stool matrixes, substantial variability in RelAbund was observed (CV ~100%). Since the internal standards were uniformly added to all samples, this variability was directly attributed to compositional measurement distortion. In contrast, the NormAbund metric exhibited low variability for both repeated analyses of a single stool composition and between the 5 different stool matrixes (CVs ranged from 2% to 11%). Generally, lower CVs were observed for higher internal standard DNA concentrations (which accounted for ~3% of all reads).
- <u>Constant sample compositions with varied actual abundances:</u> By systematically diluting stool with Tris-EDTA buffer (20% stool – 100% stool), the actual abundances of all native taxa were systematically varied while the sample composition remained unchanged. As predicted, RelAbund measurements did not reflect the known changes in actual abundance, while the NormAbund metric produced statistically significant correlations for hundreds of native taxa, with CVs generally < 25% for taxa observed at RelAbund ≥ 0.003%.

Conclusions: Using internal standards to calculate a normalized abundance metric significantly improved MGS analyses by accurately reflecting real changes in actual abundance across diverse sample compositions and concentrations. These findings were reproduced for both 16S and shotgun MGS analyses.

Tracking inter-host microbial transmission and its impact on microbial diversity

Morie Alpha, Naval Surface Warfare Center (NSWC), Indian Head, MD

Quantifying the contribution of inter-host microbial transmission to the health of individuals will allow biodefense to be calibrated to balance reducing the risk from infectious disease against the burden of microbiome diversity

decay. Infectious disease studies focus solely on the risk of pathogen transmission through a host population despite the obvious health impact of gnotobiosis, defined-flora animals, and antibiotic depletion of the microbiome. There are few studies examining potential positive health outcomes of transmitting host-associated microbes across a human population. In a series of studies based on animal data from wild and isolated populations, we are attempting to derive an estimate of microbial transmission within the populations and assess the impact of this transmission on the sustenance of microbial diversity. The factors governing this process are complex and the data is far from ideal, but some progress in observing the diversity decay in isolated populations and individuals has been made. Tying this diversity decay to health outcomes is an additional requirement for quantifying the contribution of microbial transmission to health. Ultimately, having some understanding of whether there are measurable health benefits from microbial transmission and their magnitude will allow the development of appropriate biodefense measures which may not come with hidden health costs.

Comprehensive Microbiome-Host Metadata Analysis for Disease State Classification

Hiranmayi Ranganathan, Andre' Ricardo Goncalves, Jose Manuel Martí, James B. Thissen, Nisha J. Mulakken, Car Reen Kok, Crystal Jaing, Nicholas A. Be ,

The human microbiome is inseparable from the human ecosystem. The study of microbiome interactions along with health metadata that influence disease states is very important for personalized treatment based on an individual's situational needs. Thus, investigation of how the microbiome and host metadata such as, sex, age, antibiotic use, body-mass-index, etc., impact disease state is crucial for precisely tuning and improving human health. To harness the promise of microbiome science for the future of biomedicine and healthcare, we must develop an enhanced capacity to infer clinical impacts of microbe and host metadata interactions.

Traditional microbiome studies use a single study, associate with a phenotype, and use this in isolation to drive motivation for a clinical trial. The complexity of this mechanism calls for the analysis of many microbiome studies together when linking microbial profile to a phenotype and reproducible testing to discover microbiome-related biomarkers for specific diseases.

With increasing attention to the human microbiome, sufficient data now exist for building predictive systems for the microbiome. However, existing studies have not investigated the importance of including host metadata during training to improve predictive power. In this study, we use microbiome and health metadata from 75 different datasets and use machine learning to analyze the microbiome and host metadata relations.

We present an in-depth analysis of the metadata features in a macro (across studies) and micro (within each study) level. The metadata consists of 17 informative features that when added to the microbiome data has shown to increase the predictive capability of the machine learning model. Missing metadata values across studies are imputed using multiple imputation techniques and the results are compared. This study will be an important step towards using microbiome and host metadata as a gatekeeper for diseases like sepsis, wound healing, GI disease, viral infections, and efficacy of treatments.

Isolation and Identification of Copper-Tolerant Fouling Communities

Sara M. Tuck[1], Melissa Kardish[1], Gary Vora[1], Katherine J. Franz[2], and Kenan P. Fears[1]

[1] Center for Bio/Molecular Science & Engineering, U.S. Naval Research Laboratory, 4555 Overlook Ave SW, Washington, DC 20375, USA

[2] Department of Chemistry, Duke University, Durham, NC 27708, USA

POC Email: sara.tuck@nrl.navy.mil

Accumulation of biofouling on submerged surfaces is a foundational problem for maritime transport and human health. Biofouling build-up increases the drag coefficient, fuel consumption, exhaust emissions, and operational

costs. Traditionally, biofouling is inhibited by the application of antifouling coatings, the most popular of which contain copper. In an attempt to deter the settlement of fouling organisms, copper-based antifouling coatings contain up to 75% CuO by weight. Despite these high loadings, the efficacy of these coatings is rapidly declining with the emergence and spread of copper tolerant species. Microbial communities resistant to copper have been found to form mature biofilms on these coatings, which could be altering the interfacial properties to create more favorable conditions for the settlement of a broader biofouling community. To gain an understanding of the mechanisms responsible for the loss of antifouling performance, coated and uncoated polyvinyl chloride panels were submerged at estuarine and marine field test sites and microbial communities were harvested. Collected biofouling communities were cultured and individual species were isolated and identified. Copper tolerance was assessed by re-exposing cells to copper-containing coatings and traditional antimicrobial assays to determine independent copper tolerance profiles.

Technical Session #3: Human Microbiome Characterization

Negative Energy Balance Alters Gastrointestinal Function and Gut Microbiota Composition During Strenuous Physical Activity

Cassandra Suther[1,2], Adrienne Hatch-McChesney[1], Jillian T. Allen[1], Nabarun Chakraborty[3], Alex Lawrence[3], George Dimitrov[3], Candace Moyler[3], Aarti Gautam[3], Rasha Hammamieh[3], Jess A. Gwin[1], Lee M. Margolis[1], Stephen R. Hennigar[4], James P. McClung[1], Stefan M. Pasiakos[1], J. Philip Karl[1]
[1]U.S. Army Research Institute of Environmental Medicine, Natick, MA
[2]Oak Ridge Institute for Science and Education, Oak Ridge, TN
[3]Walter Reed Army Institute of Research, Silver Spring, MD
[4]Pennington Biomedical Research Center, Baton Rouge, LA

Objective: Changes in gastrointestinal (GI) permeability and gut microbiota composition can occur during military training characterized by high physical activity and negative energy balance. The impact of energy balance on those responses is not known. This study aimed to determine the effects of altering energy balance during a period of high physical activity on GI permeability and the gut microbiome.
Methods: Ten healthy men (22 ± 5 yr) participated in a randomized, crossover study that included 12 h of baseline testing (BL) and then two 72 h periods of high physical activity (~4500 kcal/d energy expenditure) simulating sustained military operations (SUSOPS). During SUSOPS, volunteers were fed controlled diets designed to either maintain energy balance (BAL; 689 ± 852 kcal/d) or create an energy deficit (DEF; -2047 ± 920 kcal/d). Each SUSOPS phase was followed by 7 d of recovery (BAL-R, or DEF-R). Gastrointestinal (GI) transit time and pH (SmartPill), GI permeability (dual saccharide absorption test [DSAT], serum LPS binding protein [LBP]) and fecal short chain fatty

acid (SCFA) concentrations were measured during BL, DEF, and BAL. Gut microbiome composition (metagenomic sequencing) was measured during all five time points (BL, DEF, DEF-R, BAL, and BAL-R).
Results: Species-level microbiome α- and β-diversity did not differ between conditions. The relative abundance of several SCFA producing bacterial species, including *Bifidobacterium* spp., *Blautia* spp., *Mediterraneibacter* spp., *Dorea* spp. and *Faecalibacterium* spp., were lower during DEF versus BAL, but reverted back to baseline measurements during both recovery phases ($q \leq 0.25$). Additionally, 8 methanogenic archaea were lower in BAL compared to DEF ($q \leq 0.25$). Microbial gene pathways that may be related to intestinal permeability or are suggestive of nutrient insufficiency also differed between conditions (Effect size ≥ 1.0, $q \leq 0.25$). GI permeability measured by DSAT was higher during DEF versus BAL (17% [5, 32]) and BL (15% [2, 30], p = 0.03), while LBP increased 27% ([1, 53], $p < 0.001$) during PA independent of diet. Colonic transit time was longer during DEF versus BAL (mean difference: 768 min [95% CI: 364, 1173]) and BL (693 min [307, 1080]; p = 0.03). Colon pH and fecal SCFA concentrations did not differ between conditions.
Conclusions: Failure to increase intake to maintain energy balance during short-term periods of high physical activity may adversely impact GI function and gut microbiota composition.
Funding Sources: MRDC & JPC-5. Views are the authors' and do not reflect Army, DoD, or Government policy.

The effect of changes to the gut microbiome on health and performance

Sarah Harding and Emily Parish

The community of microbes that reside in our GI tract, our gut microbiome, is affected by a range of environmental, psychological and lifestyle factors including diet, antibiotic treatment, physical fitness and acute/chronic stressors. There is evidence to suggest that specific components of the gut microbiome are mediators of aspects of health and performance, including disease susceptibility, cognitive/physical states and immune responses. Defence and security personnel are exposed to increasingly multifaceted stressors, which challenge their health and performance limits, particularly when performance is required to be sustained over long periods of time, conducted in stressful and challenging environments and on overseas deployments. It is widely known that diarrhoeal disease is a significant cause of Disease Non-Battle Injury for deployed military personnel, with self-reported diarrhoeal rates over two deployments at approximately 40%, with an average of 4 underperforming days per episode. Understanding how the gut microbiome responds and adapts to military stressors may enable a non-invasive approach to build resiliency to negative health states, battlefield stress, as well as the potential to optimise the cognitive and physical performance of military. Having a better understanding of the gut, the gut microbiome, and its effects throughout the body would enable a pre-positioning in advance of a deployment which could reduce lost days, improve effectiveness, enhance survivability, and increase lethality. In addition, such findings could be exploited by a civilian population, who also suffer from the burden of GI distress—such as traveller's diarrhoea—and suboptimal performance states.

© Crown copyright (2023), Dstl. This material is licensed under the terms of the Open Government Licence except where otherwise stated. To view this licence, visit http://www.nationalarchives.gov.uk/doc/open-government-licence/version/3 or write to the Information Policy Team, The National Archives, Kew, London TW9 4DU, or email: psi@nationalarchives.gov.uk

Six Years of Longitudinal Veteran Microbiome Sampling, Major Findings, and Lessons Learned: The United States-Veteran Microbiome Project (US-VMP) Study

Andrew J. Hoisington, Christopher E. Stamper, Kelly A. Stearns-Yoder, Teodor T. Postolache, Christopher A. Lowry, Lisa A. Brenner

The microbiome field has experienced unprecedented growth in the past decade. Researchers have shown the microbiome is impacted by external factors (e.g. diet, gender, age, environmental exposures, etc) and has been

implicated in many physical and mental health conditions, including anxiety, mood, and trauma- and stressor-related disorders such as posttraumatic stress disorder. United States (US) military Veterans are a unique population in that their military-related exposures can have consequences for both physical and mental health, but the microbiome of this population has been understudied. To rectify that knowledge gap, six years ago the Military and Veteran Microbiome Consortium for Research and Education (MVM-CoRE) was formed under the Rocky Mountain MIRECC with collaborators at the University of Colorado, University of Maryland, and the Air Force Academy. The first project undertaken by MVM-CoRE was the US Veteran Microbiome Project (US-VMP), with a model to serially collect microbiome and health-related data from those seeking care within the Veterans Health Administration. The on-going research effort has collected over 6,000 fecal, oral, and skin microbiome samples from 484 participants. Using this study, we have identified important factors associated with primarily the fecal microbiome to include traumatic brain injury, alcohol use disorder, post-traumatic stress disorder, diet, social equity, homelessness and medication usage. The long-term sampling effort has also enabled within-person analysis of changes over time, to include pre- and post-COVID oral and fecal microbiome samples from 32 participants. In addition, the US-VMP study has led to knowledge on sample stabilization methods, differences between sequencing centers, and the use of mock communities for quality control in microbiome processing. This presentation summarizes the major findings from US-VMP and our lessons learned during the six year study.

The two-way interactions of travelers' diarrhea and the gut microbiome

Zachary Liechty[1], Arianna Baldwin[1], Blake Stamps[2], Adrienne Hatch-McChesney[3], J. Philip Karl[3], Michael Goodson[1]

1 711th Human Performance Wing, Air Force Research Laboratory
2 Materials and Manufacturing Directorate, Air Force Research Laboratory
3 Military Nutrition Division, US Army Research Institute of Environmental Medicine

Personnel deployed to new environments are commonly exposed to pathogens which can result in impaired health and performance, reducing their effectiveness. The most frequently reported health issue amongst deployed personnel is diarrhea. Diarrhea can afflict over 50% of deployed personnel at some point during deployment, with the majority of those affected experiencing multiple episodes. While the probability of experiencing diarrhea can be somewhat attributed to lifestyle choices while deployed (i.e., handwashing, eating MREs over local economy, etc.), these lifestyle choices do not fully explain susceptibility to diarrhea inducing agents. Another factor that could influence diarrhea susceptibility is the composition of the deployed personnel's gut microbiome. Microbe-microbe interactions could inhibit or enhance the activity of a diarrhea-inducing pathogen, leading the deployed personnel to potentially harbor a "resistant" or "susceptible" microbiome. To investigate this possibility, we surveyed the gut microbiomes of personnel from 25th Combat Aviation Brigade (Wheeler Army Airfield, Hawaii) before and after the participation in Exercise Salaknib 2022, an approximately 2 month deployment to the Philippines. We additionally collected information regarding the dietary and lifestyle habits prior to and during deployment. We have found a distinct subject-dependent shift in the microbiome after deployment compared to before. Furthermore, some of the changes in relative abundance of specific ASVs (amplicon sequencing variants) were dependent on whether the host experienced diarrhea or not, suggesting diarrhea events could have long term specific impacts on microbial composition. Furthermore, several ASVs from the pre-deployment samples were found to be positively or negatively associated with experiencing diarrhea while deployed. Some of these ASVs could make promising targets to be used as a basis for prophylactic treatment in deployed personnel to reduce the incidence of diarrheal disease during and after deployment.

Disclaimer: Authors' views not official U.S. Air Force, Army or DoD policy.

Electromagnetic Field as a Wake-up Signal for Sleeping Bacteria

Anne McCarthy (Presenter), Seung Oh (PI), Hyunjung A. Kim (Co-PI), Dale Landas, Keongsub Jeung, Yin Song, Dave Walker, Ben Masters, Kyoo Jo and Don Cropek

A key challenge to be overcome in the development of advanced multifunctional living materials is to trigger the activation of cellular functions of viable living organisms after extended storage outside of the laboratory under unpredictable, harsh conditions. Bacterial spores are some of the most resilient lifeforms on Earth and would be ideal for use in living materials; however, they are inherently highly resistant to most potential re-activation triggers, including heat, mechanical perturbation, and visible and UV light. In this project, we will investigate the application of electromagnetic fields (EMFs) as a germination trigger of Bacillus subtilis spores. While there is some research showing EMF in the gigahertz range as a germinant of bacterial spores, and more extensive research studying EMF as a sterilization technique for killing spores, little is known about the mechanism of EMF acting on spores or a complete picture of which EMF ranges can germinate spores. We've planned a systematic approach to apply different wavelengths and powers of EMF to B. subtilis spores, and to use genetic engineering, computer simulation, and lipid vesicles for temporal control of nutrient availability to uncover the mechanism of EMF-induced germination. If successful, understanding the mechanism of EMF-induced germination could lead to the creation of resilient living materials that can be activated by remote stimulus, which would have potential use in bioproduction, pollution remediation, surface concealment, and unconventional countermeasures, to name just a few applications across the Army Corps of Engineers areas of Civil Works, Infrastructure, and Operational science and engineering.

Fighting Pseudomonas aeruginosa Wound Infections with an Engineered Skin Microbe

Caleb Shin[1], J. Jordan Steel[1], Victoria Morrison[1], Vaughn Litteral[2], Camilla Mauzy[2], Kristi McElmurry[1]

[1]Department of Biology, United States Air Force Academy, Colorado Springs, USA
[2]711 Human Performance Wing, Air Force Research Laboratory, Wright-Patterson Air Force Base, Ohio, 45433, USA

Despite remarkable advances in prevention, wound infections continue to threaten U.S. military members in healthcare and combat settings, especially as bacteria gain increasing resistance to antibiotics and drug treatments. Pseudomonas aeruginosa (P. aeruginosa) is a particularly resistant strain of bacteria commonly found in the environment. It can cause infections and even death, which makes finding alternatives to P. aeruginosa antibiotics an urgent matter.

Prescribed preventive measures against P. aeruginosa infections are not always feasible in military settings, and

they are becoming less effective as the bacteria builds drug resistance. Antibiotic resistance and constrained access to care during military operations necessitate development of P. aeruginosa infection prevention and treatment alternatives. Therefore, this project aims to engineer a skin microbe to prevent infections caused by P. aeruginosa during wound healing. This novel approach to combating P. aeruginosa will also aid in preserving the efficacy of existing antibiotics.
We intend to provide a microbial countermeasure to P. aeruginosa to help prevent and treat wound infections in combat and hospital settings via a three-pronged approach. (1) Identify a P. aeruginosa target protein and genetically engineer E. coli to secrete a nanobody to for it. (2) Demonstrate nanobody excretion from E. coli and determine nanobody-P. aeruginosa interactions using classical molecular biology techniques. (3) Evaluate nanobody capability to inhibit P. aeruginosa growth and activity by killing and biofilm eradication concentration assays.
This project has the potential to deliver a microbial method for fighting P. aeruginosa to improve wound healing in combat and health care environments. It could also set the foundation for producing antibiotic alternatives for other pathogens.

Melanin in the Matrix: Transmission of Data Across Melanized Fungal Tissue

Robert M Jones[1], Dr. Robyn A. Barbato[1], Randall W. Reynolds[1], Isaac J. Tate[2], Dr. Alison K. Thurston[1]

[1]United States Army Corps of Engineers, Engineer Research and Development Center, Cold Regions Research and Engineering Laboratory
[2]University of California Riverside

Biological tissues can potentially serve as a medium for data transmission allowing for the localization of data streams to living organisms or biological sensors. In the field of intrabody communication for example, the goal is to turn the human body into an isolated communication network utilizing the individual's tissues as a transmission medium. This, however, comes with many concerns for human health as the deleterious impacts of continuous current through human tissue are not fully understood or apparent. There may be solutions in alternative biological tissues which do not bear the same concerns such as fungal and plant tissues. Fungal tissues in particular can share many of the same complexities and properties of human skin (i.e. heterogenous, anisotropy, multilayered composition) but additionally, some fungus contain a layer of melanin (a reported conductive biomolecule) which may aid in signal conduction. Here we investigate the data transmission properties of melanized fungal tissues by subjecting them to a bit rate stream assay. Pseudo Random Bit Sequences (PRBS) of increasing bit rate (1kbps-500kbps) were propagated through replicate fungal tissue samples and compared to a synchronized uninterrupted duplicate PRBS which served as a reference. The bit error rate (ratio of incorrectly received bits to total bits) was calculated for each rate. We found that as the bit stream rate increased the bit error rate rapidly increased with an average error rate of 34% at 500kbps. However, between 1kbps-100kbps the error rate is an average <10%. This range is suitable for low resolution audio transmission suggesting an applicability for the fungal tissue in that transmission space. However, it should be noted that this is a raw uncorrected signal and contemporary techniques for data correction such as forward error correction could accommodate for a higher error rate and increase the usable stream rate range for the fungal tissue.

The Effects of Bacteriophage Therapeutics for *Pseudomonas aeruginosa* infection on the Microbiome in a Model Organism

Cadet Katie Hebert ('24), Cadet Aidan Tran ('25), Dr. Caroline Amoroso, Dr. Chris Kovacs, LTC Andrew Kick

Phage therapy is a promising approach for treating multi-drug resistant bacteria, specifically a pathogen of high concern to the Department of Defense (DoD), *Pseudomonas aeruginosa,* due to its prevalence in military hospitals, infection of wounded service members, and multi-drug resistant strains. Phage therapy's effectiveness against *P. aeruginosa* has been demonstrated repeatedly in animal models and clinical usage; however, greater clarity is

needed on the possible side effects of phage therapy to include the effects on a patient's microbiome. *Caenorhabditis elegans* is an excellent model organism to investigate this question: 1) *P. aeruginosa* is pathogenic, 2) *C. elegans'* microbiome can be humanized, and 3) Lytic phage can experimentally prevent *P. aeruginosa* infection in *C. elegans.* At USMA, our researcher scientists and cadets have isolated and purified three distinct lytic phage capable of killing multi-drug resistant *P. aeruginosa*. *C. elegans* and pathogenic bacteria research is also ongoing. During this academic year, the Cadet research team will establish our *C. elegans* and *P. aeruginosa* infection model and protocols for subsequent phage therapy. Different initial microbiomes will be established and evaluated: To characterize the microbiome, molecular techniques will be conducted utilizing Next Generation Sequencing, culture techniques to purify colonies and characterize individual bacterial species, LC/GC-MS to identify metabolomics and metaproteomics, and extensive modeling is required to understand microbiome changes and is available through collaborations with the USMA Department of Math. This cadet research project is anticipated to occur over the next three academic years and enable them to build critical problem-solving and technical leadership skills, demonstrate research success, and be inspired as leaders of character and ultimately the next generation of DoD S&T decision-makers.

Technical Session #5: In vitro and in vivo microbiome models

Skin μbiome Reconstruction for Assessment of Cutaneous Effects (SURFACE)

Else Vedula, Vidhya Vijayakumar, Angela Maloney, Tyler Crawford, Jordan Hurlbut, Elizabeth Wiellette

Human skin is home to a thriving and diverse microbiome community that is causative and reactive to systemic level changes in the body, including disease- and environment-induced damage. As the most exposed barrier between the environment and the human body, these microbial communities serve critical roles in healthy states, are compositionally altered in disease states, and lend insights to the interactions between skin microbiota and other bodily systems. Like the gut microbiome, millions of bacteria that reside on the skin play both a defensive role against invading pathogens and an educational role to host immunity. Current tools for studying these processes are incomplete in that they lack the tissue complexity (in vitro) created by the presence of a complex microbiome community, or throughput and relevance (in vivo) required for efficient evaluation. Furthermore, a robust and stable testbed for engineered microorganisms is lacking.
Here, we present an in vitro model of the human skin microbiome comprised of a 5-strain consortium co-cultured with human skin tissue. Human keratinocytes were cultured onto Transwell inserts and differentiated at ALI for approximately 7 days prior to the inoculation of the 5-strain microbial consortium. Bacterial strains were individually prepared and mixed at adjusted volumes to achieve inter-strain ratios. Results show that at least two skin donors demonstrate skin morphology and strong barrier function in vitro while supporting microbiome for 7 days. Established readouts include histology, immunofluorescent staining, gene expression, and transepithelial electrical resistance (TEER), as well as relative strain abundance to characterize microbiome stability over time. Inoculation ratios between bacterial species is correlated to tissue morphology and viability. Microscopy and histology show complex tissue morphology and presence of microbial colonies on the surface of the tissue. Current and future work aims to further characterize commensal nature of consortium strains, investigate impact of disease conditions with and without consortium, build on tissue complexity by introducing higher level tissue structures, and introducing immune components. We envision this model system as a test bed to evaluate the influence of commensals on host biology, the influence of external environment on microbiome stability, the role of commensal microbes in wound healing, and the influence of the microbiome on infectious disease pathology.

Evaluation of Gut-Microbiome, Behavioral, and Physiological Responses to Acute Traumatic Psychological Stress: A Rat Model Study

James DeMar[1,2], Ryan Dennett[1], Nela Ida Crespo Rosales[1], Stephen Butler[1], Matthew Rusling[1], Allison

Hoke[1], Aurian Naderi[2], Kollin Sharpes[2], Abraham Han[2], Cooper Almarode[2], Emily Scott[2], Rachel Taylor[2], Emily Lowery-Gionta[2], Aarti Gautam[1], and Rasha Hammamieh[1]

[1]Medical Readiness Systems Biology Branch, [2]Behavioral Biology Branch / Performance Assessment and Chemical Evaluation Laboratory, Center for Military Psychiatry and Neuroscience Research, Walter Reed Army Institute of Research, Silver Spring, MD

INTRODUCTION: Acute stress reactions (ASRs) are linked with a sudden onset of extreme anxiety (e.g., tachycardia and sweating) and dissociation (e.g., amnesia and daze). These observed symptoms stem from overwhelming negative physical or psychological stimuli called traumatic stressors, where behavioral changes manifest within 24 hours post-exposure. Within days, unabated ASR symptoms can lead to the development of Acute Stress Disorder (ASD); and in turn, especially under repeated traumatic stress, can progress to Post-Traumatic Stress Disorder (PTSD) that is multifaceted in detrimental sequela and treatment resistant. Of US Soldiers engaged in combat operations, 12-25% proceed to develop symptoms of PTSD, and it is estimated that much greater numbers suffer from ASRs or ASD. Previous studies suggest that there is an interconnection between stress-related responses and its regulation by the gut-microbiome-brain-axis. This communication was shown to be bidirectional and is comprised of three parallel and interacting channels (i.e., immune, endocrine, and nervous signaling mechanisms). Presently, there are limited biological interventions which have evidence showing reduced progression from ASD to PTSD; and the antidepressant class of medication (e.g., SSRIs), which have been the most clinically investigated, has shown very limited efficacy for this indication. Thus, our study's aim is to provide a greater insight into the complex interplay between the gut-microbiome-brain-axis and brain health in hopes to assist in designing better treatment management strategies for victims of traumatic exposure.

MATERIALS and METHODS: To investigate this, adult (2 months-old) male and female Sprague Dawley rats (n = 12, each) were directly exposed to a continuous series of intense psychological traumatic stressors, i.e., a snake, ferrets, inescapable foot shocks, and then forced immersion underwater. Stressed animals were also singly housed to exacerbate the stress exposure effects. Equivalent sham animals were done, but only exposed to handling procedures and pair housed. All animals were then subjected to a recording of their "anxiety" related behavioral responses at 24 hours post-exposure, using elevated plus maze, open field, and acoustic startle tests. Newly excreted fecal pellets were also collected (i.e., quick frozen) from the animals at a baseline of 1 day prior to exposure and then at 24 hours afterwards. Microbiome evaluations of the intestinal bacterial populations within the fecal samples were carried out using 16s ribosomal RNA sequencing. Fecal microbiome data was processed using QIIME2, R, and PiCRUST2 software. Likewise, tail blood samples were collected immediately following the last stressor (i.e., immersion underwater) and then again at 24 hours post-exposure. Systemic gene expression was evaluated using transcriptomic assays of mRNA extracted from the whole blood samples, which had been treated with intracellular RNA stabilizers just prior to quick freezing. The animals were then euthanized at the 24-hour post-stress exposure mark and their brains, hearts, and plasma collected. As an initial examination of traumatic stress effects on vital organs, the hearts were immersion fixed in paraformaldehyde and processed for histopathology evaluation using microscope slides containing longitudinal sections stained with hematoxylin and eosin (H&E) and Masson's Trichrome (MT), to assess cardiomyocyte perturbations and connective tissue cell formation, i.e., scarring.

RESULTS: Behavioral testing at 24 hours following acute traumatic stress exposure versus sham conditions showed strong indications of "anxiety" related impairments to be present in both the male and female rats, with a significant ($p < 0.05$) detrimental response found on the acoustic startle test in the latter. Consistent with these behavioral findings, there was also distinct alterations in both sexes of the intestinal (fecal) bacterial microbiome, likely due an intense release of stress hormones. While the microbiome alpha-diversity was surprisingly found to be greater following stress, especially in males, the pre- and post-stress groups exhibited compositionally different microbiomes, as seen in separate beta-diversity clustering of bacteria taxa identities according to changes in abundance. Male and female rats alone also showed distinct beta-diversity clustering from each other with alterations in absolute composition. Thus, this implies that the host's body may quickly respond to stress with increases in the different types and densities of gut-microbiomes present, but in a sex dependent fashion. Interestingly, in both sexes, pathogenic bacteria strains were increased, i.e., especially the Desulfovibrionaceae

family, which could trigger production of immune response factors (e.g., cytokines) that further impair the stress resistance of the brain and other vital organs. Likewise, preliminary histopathology of the hearts suggested in some animals there was occurrence within the muscle tissue of early-stage lesions and/or aggravation of pre-existing ones, i.e., isolated pockets of cardiomyocyte degeneration and accompanying immune cell infiltration. Whole blood samples are still undergoing transcriptomic analysis for systemic changes in gene expression of proteins and related biochemical pathways, which can be accredited to a direct physiological response to stress and correlated with the microbiome disturbances.

CONCLUSIONS: Our findings in rats so far suggest that even a single event exposure to acute traumatic psychological stress, within as little as 24 hours afterwards, can markedly impact the subsequent performance capabilities as well as health status of US Soldiers. Future studies in rats are needed to extend this study to explore the effects underlying earlier as well as chronic time points post-stress exposure, e.g., 6 hours and 9 days out. Overall, our goal is to help facilitate and provide a knowledge-driven unbiased panel of biomarker and physiological signatures to aid in the diagnosis of individuals with a higher vulnerability towards stress exposure and subsequent development of ASRs, ASD, and PTSD; and thus, is an essential tool for designing their precise care management. Our study indicates that monitoring changes in the gut-microbiome following stress exposure, perhaps via fecal sampling, could prove to be a clinically practical and reliable step in this direction.

SUPPORT: This work is internally funded by the USAMRDC / MOMRP.

DISCLAIMER: Material has been reviewed by the Walter Reed Army Institute of Research. There is no objection to its presentation and/or publication. The opinions or assertions contained herein are the private views of the author, and are not to be construed as official, or as reflecting true views of the Department of the Army or the Department of Defense. Research was conducted under an IACUC-approved animal use protocol in an AAALAC International -accredited facility with a Public Health Services Animal Welfare Assurance and in compliance with the Animal Welfare Act and other federal statutes and regulations relating to laboratory animals.

In vitro fermentation shows polyphenol and fiber blends have an additive beneficial effect on gut microbiota states.

Whitman, J; Doherty, L; Pantoja, I; Racicot, K; Anderson, D; Kensil, K; Karl, JP; Gibson, G; Soares, JW*
*Corresponding

Soldier Effectiveness Directorate (SED), U.S. Army Developmental Command (DEVCOM) Soldier Center, Natick, MA, USA: Military Nutrition Division, U.S. Army Research Institute of Environmental Medicine (USARIEM), Natick, MA, USA

Various polyphenol sources and fermentable fibers have shown favorable effects on gut microbiota and microbiota-derived metabolites; however, few studies have investigated whether combining fermentable fibers & polyphenol sources may have antagonist or additive effects. Using an in vitro fermentation model simulating the physiological conditions of the ascending colon, fermentation vessels containing a nutrient-rich medium supplemented with polyphenol (PP) and fiber (FB) blends or their combination (PPFB) were seeded with pooled human fecal samples derived from 30 healthy volunteers on a habitual western diet. Fermentation aliquots were taken at 0, 5, 10 and 24h after inoculation to examine the effect on gut bacteria growth dynamics and metabolite production. Data was analyzed for statistical significance through a 2-factor repeated measures ANOVA with time, treatment and their interaction treated as within subjects factors. Upon a significant (p </= 0.05), 1-way repeated measures ANOVAs were run for between time and treatment-dependent comparisons, respectively. (PP) and (FB) blends independently lead to significant increases in the relative abundance of select beneficial taxa, namely Ruminococcus bromii, Bifidobacterium spp., Lactobacillus spp. and Dorea spp., quantified through qPCR. The benefits of both PP and FB blends are seen in PPFB. Total SCFA concentrations, identified through GC-FID, increased with fermentation time, with significant differences relative to the control evident in PPFB and FB, with minimal effect by PP and fecal only control. FB supplementation led to significant decreases in concentrations of indole, and ammonia, which are associated with proteolytic fermentation and been linked with negative gut states. The PP had limited influence and PPFB resembled FB outcomes. Increased antioxidant capacity of PPFB, relative to

the control, was driven by the presence of the PP, with FB having minimal effect. Overall, the combination of both blends, rather than showing any antagonistic or synergistic behavior, provided an additive effect on bacterial abundances and metabolite production.

Integrative Machine Learning and Bioengineered Platforms for Prediction of Military Health from the Human Microbiome

Andre Goncalves, William F. Hynes, Lindy Jang, Claire Robertson, Michael Triplett, Hiran Ranganathan, Jose Manuel Marti Martinez, James Thissen, Car Reen Kok, Monica Moya, Aubree Hinckley, Camilo Valdes, Haonan Zhu, Erica Bowers, Feliza Bourguet, Nisha Mulakken, Michael Morrison, Kyra Henderson, Mariam Mohagheghi, Aram Avila-Herrera, Crystal Jaing, Nicholas A. Be

Background: The human microbiome is a target with tremendous potential for improving military service member health and resilience. Microbiome-centric features could serve as targets with diagnostic/prognostic value for health and resilience; however, integrated computational and experimental test beds are needed to advance such solutions.
Materials and Methods: We constructed a platform that addresses these needs by establishing 1) a high-performance computing pipeline for generating metagenomic microbiome profiles, 2) a multi-task machine learning framework for predicting microbiome features relevant to human health, and 3) a bioengineered system for hypothesis testing. We trained and tested these computational and experimental systems using a range of datasets reflecting military health relevant applications.
Results: Our data fusion pipeline systematically curates metadata, paired with a metagenomic classification pipeline enabled by high-performance computing. To accommodate the complex and heterogenous characteristics of these microbiome data, we applied multi-task machine learning (ML) to existing publicly available microbiome datasets to train models that distinguish healthy and diseased subjects. These multi-task models demonstrated improved predictive performance by training on multiple datasets simultaneously and then, leveraging shared information. To alleviate the impact of high dimensionality with limited sample size, our approach automatically selected features relevant for the task at hand, in this case, disease risk prediction. To create a system capable of validating feature prediction, we developed an in vitro intestine-on-chip. This system includes intestinal scaffolds with 3D-printed villi and crypts. When seeded on these scaffolds in combination with defined microbial species, Caco-2 intestinal epithelial cells demonstrated adhesion, proliferation, and apico-basal polarization. Conclusions: Taken together, these capabilities create an end-to-end workflow for hypothesis generation and testing. Microbiome assessment and intervention represent a next frontier for precision medicine, and we are currently employing the described models for prediction of microbiome features influencing disease states in military service members. Diagnosing and testing microbiome interventions will create opportunities to enhance resilience against threats to military health and will enable testing and evaluation of solutions for deployment-associated disease. Such solutions will have far-reaching impacts for amplifying military readiness.
This effort was supported by the Lawrence Livermore National Laboratory, Laboratory Directed Research and Development program. This work was performed under the auspices of the U.S. Department of Energy by Lawrence Livermore National Laboratory under Contract DE-AC52-07NA27344.

Poster Session Abstracts

Microbiome changes as a result of Non-Euphoric Phyto-cannabinoid Elixir 14 (NEPE-14) in burn wounds using swine model.

Joshua Stephenson[1], Allison Hoke[1], Nabarun Chakraborty[1], Kristo Nuutila[2], Anders Carlsson[2,3], Rasha Hammamieh[1] and Aarti Gautam[1]

[1]Medical Readiness Systems Biology, Walter Reed Army Institute of Research, Silver Spring, MD. [2]United States Army Institute of Surgical Research, 36950 Chambers Pass, Fort Sam Houston, TX 78234, USA. [3]Metis Foundation, 84 NE Interstate 410 Loop Suite 325, San Antonio, TX 78216, USA.

Abstract: Severe burns are one of the most common injuries in recent armed conflicts, and comprehensive care must be implemented, which includes surgical debridement, skin grafting, and rebuilding. Immediate intervention can decrease injury severity by preventing excess inflammation and improve long term healing outcomes. Cannabinoids have a potential to treat wounds, as it exerts generally anti-inflammatory properties, however, little is known about its subsequent relationship to wound healing. In the current study cannabinoid drug Non-Euphoric Phyto-cannabinoid Elixir 14 (NEPE-14) was compared to standard of care burn wound dressing used within the military, silver nylon (Silverlon®). Here we studied microbiome changes as a result of these two lines of treatments.
Methods: Forty-eight deep-partial thickness burns were created on the dorsum of four anesthetized swine (Sus scrofa domestica) using a thermocoupled burn device at 100°C. One hour following burn, biopsies from each site were collected and either NEPE-14, NEPE-14 Vehicle Control, Silverlon®, or dry gauze was placed on the wound. Wounds were assessed non-invasively for the presence of bacteria on post-burn days (PBD) 3, 7 and 14 using MolecuLight® imaging system. Swabs were collected from these wounds and assessments consisted of 16S rDNA analysis for microbial features. The data was analyzed using Qiagen CLC Workbench Version 22.0.2 and the Microbiological Genomics Module pipeline to examine the microbiological data using Amplicon-Based Analysis and OTU Clustering. Our findings were based on the factors of Alpha Diversity, Beta Diversity, Permutational Multivariate Analysis of Variance (PERMANOVA), and Differential Analysis to see whether there were any significant changes in the results.
Results: *Proteobacteria* and *Firmicutes* were the two phyla that were most prevalent, followed by *Actinobacteria* and B*acteroidete*s. The two treatments with the greatest disparities in terms of timepoint were Silverlon® and NEPE-14. These two treatments also differed from one another in terms of how prominent *Proteobacteria* and *Firmicutes* were. By day 14, the microbiome had decreased in biodiversity overall due to all 4 treatments, and the wounds had also begun to heal by that point. The wounds treated with Silverlon® were found to be the least diverse by the examination of Alpha and Beta diversity, whereas the wounds treated with NEPE-14 were found to be the most diverse. This difference, however, may be explained by the fact that Silverlon® has anti-microbial capabilities unlike NEPE-14.
Conclusion: From the results gathered, it could be concluded that there were no significant differences in the microbiome between NEPE-14 and Silverlon®. As stated earlier, due to Silverlon® having anti-microbial properties the decrease in biodiversity would be expected which led to the differences in its microbiome compared to NEPE-14. With this factor taken into consideration, the microbiome differences of these different treatments are negligible. However, through comparing timepoints, it was seen that NEPE-14 restricted the growth of *Firmicutes* even though it should not have any anti-microbial properties, thus was an unexpected result that could be further evaluated for future studies. The data will be integrated with data generated from MolecuLight® imagery.
Disclaimer: Material has been reviewed by the Walter Reed Army Institute of Research. There is no objection to its presentation and/or publication. The opinions or assertions contained herein are the private views of the author, and are not to be construed as official, or as reflecting true views of the Department of the Army or the Department of Defense. Research was conducted under an IACUC-approved animal use protocol in an AAALAC International -accredited facility with a Public Health Services Animal Welfare Assurance and in compliance with the Animal Welfare Act and other federal statutes and regulations relating to laboratory animals.

Spaceflight Induced Stress Caused Alterations in Fecal Microbial Kingdoms Including Bacteria, Virus, Fungi, Protozoa and Archaea

Alexander Lawrence[1,4], Allison Hoke[1], Nicholas Gary[1,2], Aarti Gautam[1], Melissa A. Kacena[3], Rasha

Hammamieh[1] and Nabarun Chakraborty[1]

[1]Medical Readiness Systems Biology, CMPN, Walter Reed Army Institute of Research, Silver Spring, MD [2] Geneva Foundation, Tacoma, WA [3]Department of Orthopaedic Surgery, Indiana University School of Medicine, Indianapolis, IN [4]ORISE, Walter Reed Army Institute of Research, Silver Spring, MD, USA

Emerging studies found that spaceflight caused a wide range of physiological deficiencies. The Microbiome is known to contribute to host immunity and energy biosynthesis, and studies have found that spaceflight alters the function and abundance of the microbial community which may lead to dysbiosis. The aim of this research is to examine the effects of spaceflight upon the entire fecal microbiome within bacterial, archaea, virial, fungi, and protozoic kingdoms. Male C57BL/6J mice 6-8 weeks of age were used; 10 underwent surgical segmental Bone Defect under general anesthesia, and given analgesia post-operatively, 4 days prior to launch where half were housed in rodent habitats on the International Space Station (ISS) for 4 weeks, and the other half housed on ground under identical housing and experimental conditions. Also, 5 healthy mice were housed in the ISS and ground for 28 days as the baseline. Colon contents were used for shotgun sequencing following the Illumina TruSeq DNA Nano protocol on an Illumina HiSeq4000 platform. Sequenced reads were trimmed, normalized, and mapped using Bracken with bacteria, virus, fungi, protozoa and archaea databases to generate their operational taxonomic units. Mapping was done on the ERDC Onyx server using their standard and high memory nodes. Alpha and beta diversity profile, PERMANOVA, & differential expression of individual taxonomic ranks from each kingdom were calculated based on microgravity, wound, and the combination of both factors. Shannon, Simpson, and Chao1 indexes calculated the alpha diversity, while Bray-Curtis, Jaccard, Euclidean, and Manhattan indexes calculated beta diversity.At the species level: 833 bacteria, 55 archaea, 20 fungi, 0 protozoa, and 0 virus were significantly differentially expressed by drug, altitude, or both with a q value ≤ .6. Several significant findings were found from the PCoA and PERMONAOA using Euclidean, Jaccard, Bray-Curtis, and Manhattan. No Viral or protozoic species were found to be significant. The study found significant changes in the microbiome of spaceflight samples. The largest species diversity impact was seen in fungal populations. Alpha values rose for some flight individuals, and separation of flight and ground samples was seen in the varying PCoAs of some taxon. Majority of these differentially expressed taxa were found to be related to spaceflight. Further analysis of other taxonomic levels will be conducted.
Disclaimer: Material has been reviewed by the Walter Reed Army Institute of Research. There is no objection to its presentation and/or publication. The opinions or assertions contained herein are the private views of the author, and are not to be construed as official, or as reflecting true views of the Department of the Army or the Department of Defense. Research was conducted under an IACUC-approved animal use protocol in an AAALAC International - accredited facility with a Public Health Services Animal Welfare Assurance and in compliance with the Animal Welfare Act and other federal statutes and regulations relating to laboratory animals.

Growing hybrid living materials with highly ordered microstructures for exceptional mechanical properties

Alison K. Thurston, Seung Oh, Hyunjung Kim, David Walker, Yin Song, Kyoo Jo, Dale J. Landas, Logan Gonzalez, Tanner Wood, Robyn Barbato, and Donald Cropek

Microbial induced carbonate precipitation (MICP) is a ubiquitous bio-geochemical process, resulting in the precipitation of calcium carbonate. MICP has garnered attention as a potentially sustainable low carbon alternative to conventional cement. Current research predominantly focuses on a few mesophilic bacteria and temperatures exceeding 10°C in engineering applications overlooking the biological aspects driving this process. These knowledge gaps restrict the applicability of large scale MICP technology, hinder the ability to control crystal growth and strength, and limit the applicability of MICP in extreme temperature environments. To address these limitations, we are utilizing 3D printed microstructures for bacteria cultivation. Our research goals are to 1) investigate crystal growth patterns in various 3D printed predesigned structures to gain insights into the underlying mechanisms, 2)

engineer Bacillus subtilis to perform MICP in order to fine-tune the growth and orientation of the calcium carbonate crystals and 3) explore alternative microbial producers of MICP from extreme environments that could broaden the potential application of this technology. Through this multidisciplinary approach, we aim to advance our understanding of MICP, paving the way for its broader adoption in sustainable construction and environmental remediation.

Harnessing Transfer Learning for Microbiome Data Analysis

Andre R. Goncalves*, Camilo Valdes, Jose Manuel Martí, James B. Thissen,
Nisha J. Mulakken, Car Reen Kok, Crystal Jaing, Nicholas A. Be

The human microbiome, comprising trillions of microorganisms residing in and on the human body, plays a crucial role in maintaining health and in disease susceptibility. Analyzing microbiome data has emerged as a key area of research, with potential implications for personalized medicine, diagnostics, and therapeutics.
Microbiome data are complex, sparse, and limited in size, hindering training of modern predictive statistical and machine learning models. Transfer learning, a machine learning paradigm that consists in leveraging prior knowledge present in existing datasets to improve learning in new tasks, has garnered significant attention in the past few years. In this study, we delve into the evaluation of transfer learning techniques in a diverse corpus of publicly available microbiome datasets related to a variety of diseases. More specifically, we investigate two forms of transfer learning: 1) traditional transfer learning, in which a model is pre-trained on a particular *source* dataset closely related to the *target* dataset and then fine-tuned; and 2) multitask learning, in which a model for the *target* dataset is trained jointly with many *source* tasks, leveraging commonalities across all datasets. In both approaches the goal is to harness the knowledge embedded within publicly available datasets and fine-tune the models to cater to a small and crucial cohort of deployed service members.
We have curated and processed a comprehensive collection of microbiome datasets from 75 independent studies sourced from diverse populations and environments. Pre-trained models, initially trained on these datasets, serve as the foundation for the transfer learning process. Through careful fine-tuning, the models are optimized to adapt to the unique microbial composition of the deployed service members cohort, addressing the specific contextual nuances and potential confounding factors.
The evaluation process entails rigorous testing and comparison with traditional modeling approaches to gauge the effectiveness of transfer learning techniques. Key performance metrics are employed to assess predictive capabilities. Additionally, cross-validation techniques are applied to validate the model's robustness.
This study contributes valuable insights into the application of transfer learning to clinical microbiome analysis, demonstrating its potential to augment modeling capabilities in limited-data scenarios.

Longitudinal Oral and Fecal Microbiome Alterations in Veterans with New Antidepressant Monotherapy for Major Depressive Disorder

Andrew J. Hoisington*, PhD, Kelly A. Stearns-Yoder, Christopher E. Stamper, Joseph A. Simonetti, David W. Oslin, Lisa A. Brenner
*Presenting author

Major depressive disorder (MDD), a prevalent psychiatric condition, is associated with a wide range of adverse outcomes. Though antidepressants are often implemented as first-line treatments for MDD, remission and response rates remain modest. It is possible the microbiome could play a role in antidepressant treatment effectiveness. Longitudinal oral and fecal microbiome samples were obtained from ten United States Veterans, starting prior to new episodes of antidepressant monotherapy (either switching from a prior treatment or starting a new treatment) and then at weeks 4, 8, 12, 18, 24, and 30. Targeted genomic sequencing of the microbiome samples were analyzed for taxonomic and diversity changes by participants, medications, and medication class, then compared to Patient Health Questionnaire-9 (PHQ-9) scores. Taxonomic variability was observed across

participants with the individual being the main microbial community driver for both fecal and oral microbiome. In terms of the fecal microbiome, there were shifts towards *Bacteroides* being less abundant and *Blautia*, *Pseudomonas*, or *Faecalibacterium* being more abundant. In the oral samples, *Haemophilus*relative abundance decreased after starting antidepressants. Observed increases in *Blautia* and decreases in *Bacteroides*, two bacteria previously linked to immunomodulatory responses, were associated decreased PHQ-9 scores. Both oral and fecal microbial communities had a moderate shift away from baseline with antidepressant usage. In conclusion, evidential changes were associated with decreases in depressive symptoms in this study. Additional longitudinal work is required to increase understanding regarding microbiome and symptom-based changes, with a particular focus on potential differences between medication classes and underlying mechanisms.

Viral Metagenomics in Wastewater at the United States Air Force Academy: Unlocking Insights into Microbial Communities that Impact Force Health and Readiness

Jordan Steel PhD, Jordan M Goodwin C1C, Philip M Golder C1C, Amy R LeClair C1C, Michael Metchikoff MS, Darya Macmillan C1C, Allyson Peterson C2C, Armand L. Balboni MD, PhD

Wastewater is a valuable resource for studying the dynamics of microbial communities due to its complexity and the presence of diverse organisms, including viruses. Viral metagenomics, a powerful approach, has revolutionized our understanding of the viral component of environmental samples, including wastewater (Ng et al., 2011 Farcas and Moritz, 2020 Wang et al., 2022). The wastewater system at the United States Air Force Academy (USAFA) is a single point source system serving a highly characterized community of approximately 4000 college students, and another 6000 individuals including support staff, military families, and visitors. Importantly, as a military community with on-base medical facilities, we have the ability to correlate and locally track medically relevant outbreak data. We have demonstrated the first use of Virome Capture Sequence (VirCapSeq-VERT) on more than 50 temporally distinct raw wastewater samples obtained over 12-months at USAFA, to identify potential contributors to viral outbreaks in this military population. Adding VirCapSeq-VERT data to our focused RT-qPCR and clinical data tracking has allowed us to document and confirm temporal SARS-CoV-2 and Norovirus outbreaks at USAFA and has elucidated the diversity, composition, and pathogenic potential of viral communities beyond SARS-CoV-2 and Norovirus in the USAFA wastewater. With its distinct military and academic missions, monitoring the health of the base population using multiple epidemiologic tools provided critical health information to the medical community and military leadership regarding the health and readiness of the base population. This study describes the first use of VirCapSeq-VERT in support of a holistic epidemiologic approach to wastewater analysis in order to identify and track potential viral contributions to disease outbreaks at military installations.
VirCapSeq-VERT is a positive-selection augmentation to standard Next Generation Sequencing (NGS) workflow whereby the host nucleic acids, comprising the vast majority of DNA and RNA extracted from biospecimen, is discarded, and the genetic material from any vertebrate-infecting virus is retained and sequenced (Briese et al., 2015). This allows the multiplexing of individually barcoded specimen and drives down the cost of simultaneously sequencing all viruses present in a sample. VirCapSeq-VERT has been certified for use in hospitals and clinical laboratories by the New York State Department of Health and has been used in many public health and clinical research labs. VirCapSeq allowed us to identify and characterize the USAFA WW viral community without the need for cultivation and provides critical information to better inform subsequent quantitative approaches, and further support clinical observations necessary to define local outbreaks.
Self-limiting 'stomach bug' outbreaks consistent with acute viral gastroenteritis are commonly observed among the cadets at USAFA. In this young, healthy population, it is rare that anything other than oral rehydration and treatment of symptoms with antidiarrheal medication, oral hydration, and bed rest is necessary. However, these frequent outbreaks are very disruptive to a highly controlled military and academic training environment like USAFA. Current diagnostic tests including rapid testing via EIA, with PCR confirmation is generally not used as most cases resolve within 2-3 days from the onset of symptoms. While Norovirus is commonly associated with these outbreaks due to the age cohort and very close living quarters, no work had been done using wastewater to assess

whether Norovirus or other commonly associated viruses (i.e., Astrovirus or Rotavirus) were present during such outbreaks. Our qualitative VirCapSeq-VERT data demonstrate the presence of a number of variants of all of these viruses and our quantitative PCR results for Norovirus demonstrated significant increases in the wastewater viral load consistent with the clinical observations of acute gastroenteritis outbreaks. We are in the process of confirming our VirCapSeq-VERT data using RT-qPCR and sequence specific primers to further quantify each of these viruses.

We demonstrate the ongoing value of VirCapSeq-VERT to serve as an integral part of an early warning system for potential viral outbreaks. We were able to detect the circulation of families of pathogens that commonly contribute to gastroenteritis and respiratory infections common in adults and those associated with pediatric daycare center outbreaks. Future studies using viral metagenomics data at USAFA seek to not only uncover the presence of viral genomes but also provide insights into their functions and interactions with their hosts. By analyzing viral gene content and comparing it with existing databases, we hope to help predict the potential roles of viral genes in horizontal gene transfer, nutrient cycling, and host manipulation. This information contributes to our understanding of the ecological roles of viruses in various ecosystems (including wastewater) and is applicable to military operations and force protection globally.

References:

Ng, T. F., Wheeler, E., Greig, D., Waltzek, T. B., Gulland, F. M., Breitbart, M., … & Delwart, E. L. (2011). Metagenomic identification of a novel anellovirus in Pacific harbor seal (Phoca vitulina richardsii) lung samples and its detection in samples from multiple years. Journal of General Virology, 92(6), 1318-1323.

Farkas, K., & Moritz, R. L. (2020). The potential for viral metagenomics in food and environmental samples. Metagenomics, 7(2), e108.

Wang, M., Zhao, X., Tan, W., & He, S. (2022). Viral metagenomics of the microbiome in health and disease. Current Opinion in Virology, 53, 82-89.

In vitro C2C12 Muscle Cell Model Evaluation of Probiotics on Wound Healing and Fatigue Amelioration Properties

Camilla Mauzy PhD[1], Matthew Grogg PhD[1], M. Tyler Nelson PhD[1], Patrick McLendon PhD[1,2], Mary Elizabeth Huddleston[1,2], Jason Soares[3], Laurel Doherty[3], Jordan Whitman[3], and J. Philip Karl[4]

[1]Air Force Research Laboratory, 711 Human Performance Wing, RHBBB, Wright-Patterson AFB, OH
[2]UES, Wright-Patterson AFB, OH
[3]DEVCOM Soldier Center, Soldier Effectiveness Directorate, Natick, MA
[4]US Army Research Institute of Environmental Medicine, Military Nutrition Division, Natick, MA

Exhaustive and/or unaccustomed exercise induces temporary muscle damage, which can result in delayed onset muscle soreness, loss of muscle strength and power, decreased muscle function, and impaired physical performance. Restoration of muscle function and resolution of symptoms are mediated by several diverse cell types (e.g., muscle stem cells, immunomodulatory cells, etc.) that promote then resolve inflammation and oxidative stress, remove damaged tissue, and regenerate muscle fibers. This repair outcome is particularly compromised in Warfighters, a population that frequently engages in repeated high physical activities with minimal recovery during training and combat, resulting in exercise-induced muscle damage (EIMD).

New data indicate a role for gut microbiomes to impact muscle characteristics, working through a proposed '*Gut microbiome-muscle axis*" (GMA) by which gut microbes influence muscle damage, growth, and repair through multiple mechanisms. While not as well characterized as the '*Gut-Brain Axis'*, recent studies have provided preliminary evidence on potential methods of action. It is thought that specific gut microbes can initiate changes to the muscle using both direct (modulation of immune function, nutrient absorption, and gastrointestinal health) and indirect (bacterially derived metabolites) mechanisms, although it is unclear if the mechanisms are generalized or microbe specific. While academia and biotech companies have identified certain candidate probiotics that may

reduce EIMD, the studies may not have been systematically evaluated nor characterized as to specific molecular mechanisms.
Our approach uses Army/AF-developed *in vitro* methodology to examine molecular changes in muscle initiated by private sector-generated probiotics which, while having some level of human study data for claims of muscle support or enhancement, do not have a significant understanding of mechanism or molecular data. To accomplish this, we have developed collaborative agreements with 3 biotechnology companies to evaluate four probiotics and a two-component co-culture for potential effectiveness in aiding in minor muscle damage as well as to determine its effects on muscle fatigue. Probiotics were cultured in nutrient-rich medium using the NSRDEC HEL fermentation system under conditions mimicking either colonic or small intestine domain-specific conditions. After growth, the fermentation media was separated from bacteria, creating a fermentate containing all bacterially produced metabolites/short chain fatty acids produced during growth. Each fermentate and appropriate controls were then added to transwell plates containing differentiated C2C12 mouse myoblasts, using either hypoxia or normoxia conditions. Fermentate-exposed cells were evaluated for two characteristics. First, we evaluated wound reclosure using a BioTek Autoscratch in 96 well format, with wound cell growth quantitated using a Molecular Devices ImageXpress Micro and MetaXpress high content image analysis software. Working with Molecular Devices, new methodology was developed to quantitate myotube changes by adjusting image quantification to capture stain fluorescent images and creating a new software analysis module to quantitate the specific stains. Evaluations were conducted using both therapeutic (i.e. fermentate added immediately after wounding) and prophylactic (i.e. fermentate added 24 hr prior to wounding) application models. Several controls, including positive (IGF-I) and negative (L-NAME) tests, were included. Secondly, we evaluated muscle fatigue using electrical pulse stimulation (ESP) of the cells using an Ionoptix EPS system with Hz/pulse settings mimicking exercise and fatigue. After EPS, cells were evaluated for viability (LDH, ATP), function, and morphology. In addition to probiotic fermentate testing, four purified short chain fatty acids were evaluated using both the wound healing and fatigue evaluations.
Wound closure analyses identified a slight improvement using one probiotic fermentate. However, it was noticed that myotube structure developing within the wound differed between fermentate exposures as to length and width. To allow capture of these data, we developed a new method to quantitate myotube-associated length and nuclei during the healing process. This newly developed protocol allows us to examine not only how fast the wound heals, but the cellular structure developed within the wound to understand if it was largely myoblastic cell growth (proliferation) or higher myotube concentrations, and if these characteristics (or a combination of the two) predict better muscle function after healing. While the scratch assay is well established, the myotube characterization protocol is a unique method with its first application in this study. Using the myotube characterization procedure, we saw 35-45% portion of myotubes in all treatments, but specific fermentates did have specific ranges, with one probiotic demonstrating lower myotube levels but higher myotube/myoblast ratios. The same probiotic fermentate demonstrated myotube-associated nuclei within the wound was demonstrated improved over controls after 48 hr of growth. Similar analyses using purified SCFAs indicate an increase in myotube-associated nuclei and myotube area with minimal changes in myotube/myoblast cell count. Fatigue analyses using fermentate exposed C2C12 cells did not demonstrate any optimal changes in fatigue biomarkers ATP, lactate, and LDH, nor myokine alterations. However, further refinement on examination of differentiated C2C12 + EPS myokine expression and myokine responses with/without fermentates are ongoing.
While these studies indicated minor improvement in wound healing using only one fermentate, the developed *in vitro* muscle model could be widely used to examine many effectors for their effectiveness in supporting wound healing and suppression of muscle fatigue in normal or hypoxic environments and to provide a mechanistic understanding of new therapeutic or prophylactic applications. With further refinement, we anticipate our technology will identify additional probiotic/probiotics which can be used as dietary supplements. The use of a GMA-based probiotic is a novel intervention to mitigate and promote recovery from exercise-induced injury, and its use for musculoskeletal support could be used in addition to other injury reducing methods to decrease injury, optimize performance, and, ultimately, enhance lethality.
Disclaimer: The views expressed are those of the authors and do not reflect the official guidance or position of the Air Force, the Army, the Department of Defense, or the United States (U.S.) Government.

DISTRIBUTION A: Abstract has been cleared as Case Number: AFRL-2023-0448 on 27 Jan 2023.

Deep Embeddings for Characterizing Metagenomic Abundance Profiles in Longitudinal Studies

Camilo Valdes , Andre R. Goncalves , Jose M. Mart´ı , James B. Thissen , Nisha J. Mulakken , Car Reen Kok , Crystal J. Jaing , and Nicholas A. Be

Metagenomics is the study of the combined genetic material found in microbiome samples, and it serves as an instrument for studying microbial biodiversities and their relationships to their host environments. Profiling a microbiome is a critical task that tells us what microorganisms are present, and in what pro-portions; this is particularly important as many human diseases and environmental disasters are linked to changes in microbial compositions.

Creating, interpreting, and understanding microbial community abundance profiles produced from mi-crobiome samples is a challenging task as it requires large computational resources along with innovative techniques to process datasets that contain terabytes of information. Deep learning methods are a proven technique for working with microbiome data sets because of their ability of processing large amounts of data and drawing out meaningful patterns. In microbiome analyses, deep neural networks usually operate on raw abundance counts, and deep learning embeddings are abstract, low-dimensional numerical representations of the abundance profiles.

In this work we present a novel context for using microbiome sample embeddings created with a neural-driven fusion model that uses multiple complementary representations of metagenomic abundance profiles. The embeddings are not created from the abundance counts directly, but from representations that con-textualize the microbial abundance and taxonomical space. We use them to cluster and visualize the rep-resentations of microbial features related to taxonomical clades, important time-series points, and disease types. The embeddings are efficient, portable, and not method specific: they can be used in a large range of downstream analysis tasks such as classification, clustering, and visualization.

We analyzed multiple publicly available datasets (static and longitudinal), comprising over 12 K unique samples representing multiple diseases, environments, and time points. Each of the microbiome samples are represented in two complementary ways: a set of 2D images created with Hilbert Curve Visualizations (HCV), and the important taxonomic groups identified by a hierarchical feature engineering (HFE) method. The resulting embeddings are visualized using the Uniform Manifold Approximation and Projection (UMAP) method, and contextualized by their associated condition label (disease, environment) or time point.

The last couple of years have seen a rapid increase in the amount of metagenomic data that is available to researchers as low-cost, high-throughput metagenomics DNA sequencing is becoming more accessible. The complex latent properties of microbiomes embedded in community abundance profiles created by these studies are not easily uncovered using traditional representation techniques, and the distinctive patterns created by our model in low dimensional space can be used to identify important microbial species. Our work highlights the use of an embeddings model using large metagenomic datasets with static and longitudinal components, and which enable the detection of critical microbial groups at different time points and disease types.

The military gear microbiome: risk factors surrounding the warfighter

Car Reen Kok[1], Zakariae Bram[2], James B. Thissen[1], Timothy S. Horseman[2], Viseth Ngauy[2], Catherine F. Uyehara[2], Nicholas A. Be[1]

[1]Physical and Life Sciences Directorate, Lawrence Livermore National Laboratory, Livermore, CA
[2]Tripler Army Medical Center, Honolulu, HI

Introduction: Combat extremity wounds are highly susceptible to contamination from surrounding environmental material. This bioburden could be partially transferred from materials in immediate proximity to the wound, including fragments of the uniform and gear. Opportunistic pathogens that can survive on gear represent risk factors for infection following injury, especially following combat blasts, where fibers and other materials are embedded in wounded tissue. Despite such risks, no study to date has assessed the microbial bioburden present on

military gear during operational conditions of deployment or training. In this study, we utilized 16S rRNA sequencing to assess the microbiome composition of different military gear types (boot, trouser, coat and canteen) from two operational environments (Hawaii and Indonesia) across time (days 0 and 14), providing, for the first time, foundational data on the microbiome of military gear to evaluate the potential bioburden threats surrounding military service members. Overall, this study emphasizes the importance of microbial surveillance in environments of proximity to the warfighter to advance military preparedness and protect warfighter safety and wellbeing.
Materials and methods: Fifty participants were recruited from students enrolled in 25th Infantry Division's (ID) three-week Jungle Operations Training Course on Oahu, Hawaii and from 25th ID active-duty service members deployed to Indonesia for a multi-national exercise (Garuda Shield). Swab samples were obtained from the underside of boots and from coats and trousers, and samples of drinking water were collected from canteens. Baseline samples were taken upon arrival and 14 days after the start of operations. Specimens from participants that completed all sampling locations from both timepoints were selected for microbiome profile processing (28 from Hawaii and 33 from Indonesia). DNA was extracted from samples and the 16S rRNA V3-V4 region was amplified and sequenced (Illumina 2 X 300bp). Analysis of 16S rRNA sequences were carried out in QIIME2. Amplicon Sequence Variants (ASVs) were obtained using the DADA2 algorithm and taxonomic assignments were assigned based on the SILVA database. Diversity analyses of microbiome samples was conducted using phyloseq in R. Alpha diversity was measured using observed ASVs and Shannon index while beta diversity was measured using non-metric dimensional scaling (NMDS) of Bray-Curtis distances. Differential abundance analysis was carried out using DESeq2 in R. Functional predictions of microbiome samples were carried out using PICRUSt2 and gene families were assigned to KEGG Orthologs (KOs). Annotations of antimicrobial resistance genes were obtained from the KEGG database while virulence gene annotations were obtained from a published customized database of virulence-relevant KOs.
Results: We successfully profiled the microbiome of military gear under operational field environments and were able to identify the presence of various microbial risk factors. Several wound-colonizing species; Acinetobacter, Pseudomonas, Streptococcus, and Staphylococcus were found to be present in all sample types and at both study sites. In addition, microbiome diversity, stability, and composition were found to be dependent on gear type, training location and timepoint. Boot samples had the highest alpha diversity, followed by trouser and coat samples while the alpha diversity of canteen samples was significantly lower ($p<0.05$) compared to all other gear types regardless of study location, timepoint, and diversity metric. Beta diversity analysis also demonstrated that canteen samples were compositionally distinct from other gear types and were similar across study sites and timepoints. On average, canteen samples were dominated by Proteobacteria (Indonesia; 92,9% ± 11,2, Hawaii; 78.6% ± 21.1). From the NMDS plots, compositional differences were observed between Hawaii and Indonesia samples at Day 0 and Day 14 with apparent compositional shifts across time for Hawaii samples. On average, samples from Indonesia were found to be Firmicutes-dominant (trouser; 52.4% ± 17.27, coat; 50.7% ± 18.8 and boot; 31.5% ± 24.3) while Hawaii samples were found to be Proteobacteria-dominant (trouser; 52.5% ± 17.0, coat; 49.5% ± 22.1 and boot; 60.7% ± 8.3). Permutational multivariate analysis of variance revealed significant effects of study sites, gear types and timepoints along with significant interactions between all three factors ($p<0.001$). Differential abundant analyses further demonstrated that at Day 14, the number of significantly differential ASVs ($p<0.05$, log2FoldChange > 2) were higher in Hawaii samples (boot: 471, canteen: 0, coat: 479, trouser: 505) compared to Indonesia samples (boot: 96, canteen: 3, coat; 90, trouser: 98) which may reflect PICRUSt2 predicted potential antimicrobial resistance and virulence burden that may be present within the military gear microbiome. This includes antimicrobial resistance genes involved in multidrug resistance and cationic antimicrobial peptide resistance, and virulence genes involved in adherence, antiphagocytosis, invasion, iron uptake and secretion systems. Operational tempo along with environmental conditions may have influenced the differences seen in microbiome density and diversity between Hawaii and Indonesia. Students in Jungle Operations Training had limited access to laundry facilities and had a more compressed training schedule than participants in Indonesia. **Conclusions:** Our study presents novel information on the military gear microbiome in real-world operational settings along with potential risk factors that exist around the warfighter. This information can be used to guide design of antimicrobial materials and uniforms and for infection control efforts following combat blast and other injuries, thereby improving treatment guidance during military training and deployment. Further studies that integrate dense longitudinal sampling, collection of health-relevant metadata, and the surveillance of other microbial species such as fungi and virus are necessary to

fully elucidate risk factors that could be present.

Disclaimer: Any expressed opinions are the authors' alone, and do not reflect the views of any U.S. Government agency. Prepared by LLNL under Contract DE-AC52-07NA27344.

Acknowledgements: The authors wish to thank Dr. Keith Fong, Ms. Susan Reichert-Scrivner, and MAJ Kristina Thompson for sample collection in the field which made this study possible.

Metagenomic and Culture-based Characterization of the Chesapeake Bay Microbiomes

Ahren W. Jin[1], Caitlyn J. Koo[1], Logan M. Treaster[1], and Robert K. Ernst[2], Charles R. Sweet[1]

1 – United States Naval Academy Chemistry Department, Annapolis, MD 21402,
2 – University of Maryland School of Dentistry Department of Microbial Pathogenesis, Baltimore MD 21201

1 – United States Naval Academy Chemistry Department, Annapolis, MD 21402, 2 – University of Maryland School of Dentistry Department of Microbial Pathogenesis, Baltimore MD 21201

The Chesapeake Bay is the largest estuary in the United States and is of ecological, economic, and strategic importance. We are conducting a multi-year effort to determine the structure of the planktonic bacterial microbiome in both the Middle Bay (Annapolis region) and the Severn River by metagenomic characterization of whole-community genomic DNA using full genome shotgun sequencing, assembly, and analysis. These datasets demonstrate characteristic differences in the metagenomic signature of the microbiome composition by location in the summer, and a dramatic difference between the summer and winter populations. The winter microbiome is, however, more uniform by location than the summer one, a commonality that suggests the winter microbiome is repopulated each year from a common hibernal source. In addition to this metagenomic effort, we have also identified many members of a large culture collection developed concomitantly from the same water samples. Analysis of resulting 16S sequencing data yields similar observations about distribution and variation of the culturable subset of the microbiome with season and location, and suggests our culture-based understanding of the environmental microbiome is not yet saturated due to the discovery of novel bacterial genera and species endemic to the Chesapeake Bay.

Response of soil microbial communities to soil amendment, inoculation, and RDX contamination

Chris Baker, Flora Lauren, Alison Thurston, Robyn Barbato

Abstract: The explosive RDX is a common soil and groundwater contaminant on US military live-fire training ranges. RDX is recalcitrant to breakdown, causing it to accumulate over decades, posing known or potential threats to the environment and human health. Efforts to remediate contaminated soil and water by augmenting or stimulating soil microbes have met with mixed success. Several RDX-metabolizing enzyme systems have been identified in naturally-occurring bacteria and fungi. However, bioremediation efficacy is often inconsistent, possibly as a result of local edaphic conditions, interactions with co-contaminating chemicals, or poor survival of microbial inocula. In this study, we use a microcosm experiment to investigate soil microbial community composition against backgrounds of two different soils, amendment with compost, inoculation with known RDX-degrading bacteria, and RDX contamination. Amplicon sequencing revealed strong effects of soil origin and compost amendment on overall community composition. In comparison, RDX contamination and inoculation had limited effects at the community level. However, the influence of RDX on bacterial communities showed an interaction with compost treatment, suggesting that microbial bioremediation efforts might be influenced by nutrient limitations or, alternatively, by shocking incumbent microbes with inoculations of co-adapted microbial consortia.

Advancing Ice Mitigation: Exploring Antifreeze Proteins for Sustainable Freezing Prevention in Cold Environments

Elizabeth J. Corriveau[1], Shae M. Nestor[1], Lina J. Bird[2], Emily Asenath-Smith[1], Robyn A, Barbato[1]

[1]United States Army Corps of Engineers, Engineer Research and Development Center, Cold Regions Research and Engineering Laboratory, Hanover, NH, USA
2Center for Bio/Molecular Science and Engineering, Naval Research Laboratory, Washington, D.C. 20375, USA

Freezing prevention presents a significant challenge to the military in cold regions. Existing ice mitigation methods, like pneumatic boots or hot-air anti-icing systems used on commercial aircrafts, are categorized as 'active methods,' but they often entail high energy consumption or result in negative environmental impacts. Antifreeze proteins (AFPs) offer a promising alternative, as they provide anti-freeze properties at low concentrations through non-colligative mechanisms based on specific molecular interactions. This study aims to isolate environmental microorganisms living in cold regions, test whether they harbor AFP, and investigate their capacity to modify ice crystal formation and inhibit recrystallization.
Samples of ice-wedge collected from the CRREL Permafrost Tunnel in Fox, Alaska underwent 27 freeze-thaw cycles, ranging from -10°C to 15°C. From these samples, four bacteria were isolated and subsequently characterized using gram staining and 16S sequencing. To test their influence on ice crystal formation, crude lysate will be extracted from the isolates, exploring their potential use in coatings and materials for ice mitigation applications. This research contributes to the advancement of environmentally friendly and energy-efficient freezing prevention methods, with potential implications in various industries, such as frozen food and cryomedicine.

Gut microbial dynamics as a function of differential supplementation

Ida Gisela Pantoja-Feliciano De Goodfellow[1], J. Philip Karl[2], Matthew Perisin[3], Laurel A. Doherty[1], Holly L. McClung[2], Nicholes J Armstrong[2], Rebecca Renberg[4], Kenneth Racicot[1], Tobyn Branck[1], Steve Arcidiacono[1], Jason W. Soares[1]

1. Soldier Effectiveness Directorate (SED), U.S. Army DEVCOM Soldier Center, Natick, MA, United States.
2. Military Nutrition Division, U.S. Army Research Institute of Environmental Medicine (USARIEM), Natick, MA, United States.
3. U.S. Army DEVCOM Army Research Laboratory, Adelphi, MD, United States.
4. General Technical Services, U.S. Army DEVCOM Army Research Laboratory, Adelphi, MD, USA

Abstract: The human gut microbiome can be modulated by host exposure to different stressors. Diet in particular, can greatly influence the dynamics of gut microbiome composition and metabolism. In our laboratory we used an *in vitro* gut model to investigate the influence of a sudden change in diet, namely 21 days sole sustenance on the Meal, Ready-to-Eat (MRE) U.S. military combat ration on population dynamics within the gut microbiome using nutrient-rich media supplemented with saccharolytic and proteolytic substrates. Under saccharolytic conditions, fecal samples collected from individuals before and after consuming their habitual diet or only MREs for 21 days underwent 24hr *in vitro* fermentation in nutrient-rich media supplemented with RS under ascending colon domain-specific conditions. 16S rRNA amplicon, Whole Genome Sequencing (WGS) and specific statistical analysis were used to measure community composition and functional potential. 11 taxa showed differential changes in relative abundance; as an example, we can mention *Dorea spp* which notably increased in the MRE day 21 group after 10hr of exposure to starch-supplemented medium relative to the other groups. Nine Carbohydrate-active enzyme (CAZymes) showed interesting patterns due to MRE day 21 group, specifically GH13_14 significantly higher at 10 and 24hr in MRE day 21 group compared to the other groups. When adding proteolytic substrates, the goal was to determine if the MRE diet generates a community that shifts toward more proteolytic metabolic behavior, which would potentially generate more pro-inflammatory compounds. Fermentations in triplicates were conducted in

which the ratios of carbohydrate and protein substrates varied: HCLP (high carb, low protein, 75/25), CP (basal medium, 50/50), LCHP (low carb/high protein, 25/75) and P100 (100% protein, no carbs). The results of this study will help to explore functional capacities of the community as a function of MRE diet to add to human study population analyses.
Disclaimer: The views expressed in this abstract are those of the authors and do not reflect the official policy of the Department of Army, Department of Defense, or the U.S. Government.

Microbiome changes correlate with human health and performance measures during a seven-month submarine deployment.

Joanna Halford

Introduction: Submariners experience unique challenges, including a limited diet, a lack of sunlight cues to maintain circadian rhythm and vitamin D, interrupted sleep, and stress. Some of these challenges are known to disrupt the microbiome, causing ill health in the general population, including obesity, metabolic syndrome, impaired cognitive performance, and mental health issues. This raises the possibility that a prolonged submarine deployment could degrade health through adverse effects on the microbiome. Understanding whether certain microbiomes confer resiliency to deployment stressors and if/how changes in the gut microbiome during a prolonged submarine deployment impact health and performance may offer an opportunity to improve the health and performance of submariners through interventions targeting the microbiome.
This study had three aims. 1) To characterize changes in microbiome, metabolome, and proteome (specifically, fecal microbiome/metabolome, and blood metabolome/proteome), during a prolonged submarine deployment. 2) To determine whether any identified deviations were associated with changes in submariner health and performance that might impact operational readiness. And 3), To determine whether changes in diet and/or the microbiome of the built environment correlate with changes in the human microbiome.
Materials and Methods: Thirty U.S. Navy submariners, serving on a fast attack submarine (SSN), provided fecal samples, blood samples, and skin swabs; completed questionnaires relating to their diet (Block Food Frequency Questionnaire) and mood (abbreviated Profile of Mood States); and completed serial cognitive assessments (Automated Neuropsychological Assessment Metrics) before, during, and after a seven-month deployment. Objective sleep data were also collected during the deployment, using actigraphy watches (Motionlogger Micro Watch). Environmental sampling of various surfaces aboard the submarine was also conducted before and during the deployment, to coincide with the skin swabs from the subjects, to explore the effects of the environmental microbiome on the human microbiome.
Analysis of the fecal, skin, and environmental samples included DNA-based sequencing to identify the microbes present; and for the fecal samples, we also used digital PCR techniques, to determine absolute abundances of those microbes. High throughput metagenomic sequencing of the fecal samples was done to assess the functional capacity of the microbes present, and untargeted metabolomics analysis was used to assess the functional activity of the microbes. The blood samples were analyzed to identify metabolites and proteins of interest, focusing on those with established links to stress, neurocognitive function, immune function, and gut barrier function. The dietary and mood questionnaires, actigraphy data, and cognitive assessments were analyzed to identify any changes over the course of the deployment, and any correlations between those changes and changes in the microbiome.
Results: This study builds on our previous work (Stamps et al., 2022), which identified deployment-associated changes in the microbiome of submariners during an eight-week deployment on a ballistic missile submarine (SSBN). These changes were associated with changes in metabolism, changes in inflammatory blood markers, and increased fatigue. This follow-up study, on a different submarine type during a longer deployment, also demonstrated changes in the composition and diversity of the microbiome. In both studies, gut microbiota diversity was affected more by the Service member from which the sample originated than the phase of deployment. However, deployment did have a significant impact on both diversity and microbial composition, with certain species decreasing or increasing while underway. Furthermore, in this study, the observed changes in the

microbiome had not fully returned to baseline 3 months after the submariners' return to home port. Questionnaire data also demonstrated an increase in negative emotions, and a reduction in healthy eating, with increased consumption of foods associated with inflammation, during the deployment. Some of the observed changes in mood and diet significantly correlated with changes in the microbiome. Analysis of the cognitive and actigraphy data, along with the skin and environmental swabs, is ongoing.
Conclusions: Preliminary results indicate that, while the microbiome is highly individualized, deployment did significantly impact its composition. Furthermore, the microbiome of Service members significantly correlated with various aspects of mood and eating habits, suggesting that the microbiome may influence health and performance, and can potentially be altered through dietary interventions. Understanding whether certain microbiomes confer resiliency to deployment stressors and if/how changes in the gut microbiome during a prolonged submarine deployment impact health and performance will enable us to identify targeted countermeasures to improve submariners' health and resiliency and, therefore, mission readiness of the fleet.
Disclaimer: The views expressed in this abstract reflect the results of research conducted by the authors and do not necessarily reflect the official policy or position of the Department of the Navy, Department of Defense, nor the U.S. Government.
Source of support: This work was supported by DHA (Sustainment) funding Work Unit Number F1805.
Human subject research protections: The study protocol was approved by the Naval Submarine Medical Research Laboratory Institutional Review Board in compliance with all applicable Federal regulations governing the protection of human subjects. NSMRL IRB approved protocol #: NSMRL.2021.0004.
Some authors are military Service members and employees of the U.S. Government. This work was prepared as part of their official duties. Title 17 U.S.C. §105 provides that "Copyright protection under this title is not available for any work of the United States Government". Title 17 U.S.C. §101 defines a U.S. Government work as a work prepared by a military Service member or employee of the U.S. Government as part of that person's official duties.

The EPA Systems Approach to Biological Contaminants in Order to Improve Indoor Air Quality

Jordan Zambrana[1*]
U.S. Environmental Protection Agency, Office of Radiation and Indoor Air, Indoor Environments Division, Washington, DC 20460, USA
*Address correspondence to Jordan Zambrana, Email: Zambrana.jordan@epa.gov, 1301 Constitution Ave NW., Washington, DC 20460,

The quality of air indoors is impacted by all the living things in it. All the living microorganisms inside an indoor environment, and their by-products, are known collectively as an indoor microbiome. The U.S. Environmental Protection Agency's (EPA) Indoor Environments Division (IED) has recently published a new webpage focused on the indoor microbiome and its impact on indoor air quality. This new web content is a part of our role in supporting and promoting research to address identified gaps in knowledge and allow for greater dissemination of information related to the indoor environment, microbiomes, and human health. Since the biological component of the indoor environment is both impacted by and impacts all aspects of the built environment, from the microbial ecology of indoor water systems to the air a building occupant inhales, IED has adopted a systems approach.
IED webpage content promotes voluntary strategies for reducing or eliminating indoor exposures to biological contaminants that contribute to health risks in homes, schools and other non-industrial buildings. Practical strategies for improving indoor air quality and minimizing risk of exposure are discussed including such topics as *Legionella* and infectious disease transmission indoors. The effectiveness of these key strategies of source control, ventilation, filtration, and air cleaning is maximized through the application of a whole built environment systems approach.

Microbiome changes as a result of Non-Euphoric Phyto-cannabinoid Elixir 14 (NEPE-14) in burn wounds using swine model

Joshua Stephenson[1], Allison Hoke[1], Nabarun Chakraborty[1], Kristo Nuutila[2], Anders Carlsson[2,3], Rasha Hammamieh[1] and Aarti Gautam[1]

Medical Readiness Systems Biology, Walter Reed Army Institute of Research, Silver Spring, MD.
United States Army Institute of Surgical Research, 36950 Chambers Pass, Fort Sam Houston, TX 78234, USA.
Metis Foundation, 84 NE Interstate 410 Loop Suite 325, San Antonio, TX 78216, USA.

Abstract: Severe burns are one of the most common injuries in recent armed conflicts, and comprehensive care must be implemented, which includes surgical debridement, skin grafting, and rebuilding. Immediate intervention can decrease injury severity by preventing excess inflammation and improve long term healing outcomes. Cannabinoids have a potential to treat wounds, as it exerts generally anti-inflammatory properties, however, little is known about its subsequent relationship to wound healing. In the current study cannabinoid drug Non-Euphoric Phyto-cannabinoid Elixir 14 (NEPE-14) was compared to standard of care burn wound dressing used within the military, silver nylon (Silverlon®). Here we studied microbiome changes as a result of these two lines of treatments.
Methods: Forty-eight deep-partial thickness burns were created on the dorsum of four anesthetized swine (Sus scrofa domestica) using a thermocoupled burn device at 100°C. One hour following burn, biopsies from each site were collected and either NEPE-14, NEPE-14 Vehicle Control, Silverlon®, or dry gauze was placed on the wound. Wounds were assessed non-invasively for the presence of bacteria on post-burn days (PBD) 3, 7 and 14 using MolecuLight® imaging system. Swabs were collected from these wounds and assessments consisted of 16S rDNA analysis for microbial features.
The data was analyzed using Qiagen CLC Workbench Version 22.0.2 and the Microbiological Genomics Module pipeline to examine the microbiological data using Amplicon-Based Analysis and OTU Clustering. Our findings were based on the factors of Alpha Diversity, Beta Diversity, Permutational Multivariate Analysis of Variance (PERMANOVA), and Differential Analysis to see whether there were any significant changes in the results.
Results: *Proteobacteria* and *Firmicutes* were the two phyla that were most prevalent, followed by *Actinobacteria* and B*acteroidete*s. The two treatments with the greatest disparities in terms of timepoint were Silverlon® and NEPE-14. These two treatments also differed from one another in terms of how prominent *Proteobacteria* and *Firmicutes* were. By day 14, the microbiome had decreased in biodiversity overall due to all 4 treatments, and the wounds had also begun to heal by that point. The wounds treated with Silverlon® were found to be the least diverse by the examination of Alpha and Beta diversity, whereas the wounds treated with NEPE-14 were found to be the most diverse. This difference, however, may be explained by the fact that Silverlon® has anti-microbial capabilities unlike NEPE-14.
Conclusion: From the results gathered, it could be concluded that there were no significant differences in the microbiome between NEPE-14 and Silverlon®. As stated earlier, due to Silverlon® having anti-microbial properties the decrease in biodiversity would be expected which led to the differences in its microbiome compared to NEPE-14. With this factor taken into consideration, the microbiome differences of these different treatments are negligible. However, through comparing timepoints, it was seen that NEPE-14 restricted the growth of *Firmicutes* even though it should not have any anti-microbial properties, thus was an unexpected result that could be further evaluated for future studies. The data will be integrated with data generated from MolecuLight® imagery.
Disclaimer: Material has been reviewed by the Walter Reed Army Institute of Research. There is no objection to its presentation and/or publication. The opinions or assertions contained herein are the private views of the author, and are not to be construed as official, or as reflecting true views of the Department of the Army or the Department of Defense. Research was conducted under an IACUC-approved animal use protocol in an AAALAC International -accredited facility with a Public Health Services Animal Welfare Assurance and in compliance with the Animal Welfare Act and other federal statutes and regulations relating to laboratory animals.

Delayed Impact of Radiation on Meta-Organism Depends on Gender: Metageomics and Metabolomics Analysis of Mouse Fecal Samples

Matthew Rusling[1], Nabarun Chakraborty[1], Gregory Holmes-Hampton[2], Vidya P. Kumar[2], Allison Hoke[1], Alexandra Lawrence[1,3], Aarti Gautam[1], Kevin Swift[1], Rasha Hammamieh[1] and Sanchita P. Ghosh[2]

[1]Medical Readiness Systems Biology, CMPN, Walter Reed Army Institute of Research, Silver Spring, MD
[2]Armed Forces Radiobiology Research Institute, Uniformed Services University of the Health Sciences (USUHS), Bethesda, Maryland 20889-5603, United States
[3]Vysnova, Inc. CMPN, Walter Reed Army Institute of Research, Silver Spring, MD

Background: The need to build a time dependent knowledge about the nuclear radiation exposure is more pertinent than ever given the permeation of ionizing radiation in our society's framework. Not only has the threat of global nuclear events become more imminent in recent years, but the escalated handling of nuclear radiation in industrial and medical sectors also pose risks for accidental radiation exposure. Exposure to ionizing radiation causes dose-dependent health effects and are characterized as Acute Radiation Syndrome (ARS). Studies have indicated that the fecal commensals, a highly enriched and diverse community of microorganism play significant roles in combating stress; in particular, those microorganisms that colonized on or in the host can endure footprints of the stress long since the stress exposure. Taken together, fecal microbiota could be an eligible biomatrix to characterize the delayed effects radiation. The result could facilitate diagnosis an accidental exposure of radiation long time since the event takes place, and this knowledge could further inform next generation therapy. In this study, we screened fecal metagenome and metabolites. Emerging evidence suggests that the metabolomics is the converging node of multiple kingdoms; therefore, the screening of metabolites can inform the health of meta-organism that embodies the host and its resident microbiota.
Hypothesis: The meta-organism fosters the comprehensive response to sublethal radiation and gender is an important co-factor to define this response dynamics.
Objective: To find gender-/dose-/time-specific fecal metagenomics and metabolite markers and corresponding biomechanisms responding to lethal radiation.
Materials and Methods: Thirty C57BL/6j adult mice (N=15 male, N=15 female) were exposed to total body irradiation (TBIr) at doses of 7Gray (N=10) and 7.5Gray (N=10) in a single exposure event; a 10 mice sham group was handled identically. Fecal samples were collected 1 month (1m) and 6 months (6m) time since radiation (TSR) and saved at -80^{0}C until DNA extraction. Subsequently, vendor recommended set of primers isolated, barcoded and amplified the hyper-variable V3 and V4 regions of the 16S rRNA amplicon in the Illumina MiSeq platform. The de-multiplexed sequences were analyzed using QIIME2 and DESeq2. PiCrust delivered those functional networks, which were potentially enriched by microbiota. Differentially abundant taxa and functional networks were curated by 3-way ANOVA using the co-factors namely gender, time and dose. Targeted metabolomics and lipidomics assays were conducted using mass spectrometry performed on a Quadrupole Time-of-Flight (Q-TOF) Premier mass spectrometer (Waters Corporation, Milford, MA, USA). Differentially expressed (DE) metabolites and lipids were curated by 3-way ANOVA, as described earlier. Pertinent functional analysis was performed using IPA. Finally, the networks potentially enriched by meta-organisms and microbiota, respectively were integrated to map the host-microbiota bidirectional relationship in responding to sublethal TBIr.
Results and Discussion: Estimating the compositional variabilities among the microbiome communities, PERMANOVA primarily attributed the beta-diversity to the co-factors namely gender and dosimetry. Time since radiation (TSR) also had a smaller effect size. The separation between male and female cohorts was distinct in Unweighted Unifrac and Jaccard plots that suggested a differentially enriched community of microbiota between two genders. The gender specific analysis was followed. Among the male mice, TSR emerged as a significant factor to explain the beta-diversity; while among the female mice, both dosimetry and TSR emerged as significant co-factors explaining the beta-diversity.
Alpha-diversity revealed a gender-specific shift in fecal commensals within a community. Chao1, a primary metrics of richness showed a gender-specific divergence. At 6m since 7.5Gy TBIr, Chao1 significantly increased in male, but

reduced in female cohort. Shannon and Simpson diversity measure both evenness and richness, and these diversity metrics were primarily affected by the cumulative effects of dose and time.
Furthermore, we curated the major phyla to understand individual abundance across this study landscape. The abundance profile of the phyla namely *Firmicutes* and *Verrucomicrobia* varied between the genders. The shift in *Firmicutes* was primarily due to TSR and dosimetry in male and female groups, respectively. In contrast, the shift in *Verrucomicrobia* was due to the interaction between dosimetry and TSR in both male and female groups.
PCA plot of fecal metabolite and lipid profile demonstrated gender specific clustering like the metagenomic profile. Subsequent functional analysis revealed that the networks linked to lipid and amino acid metabolism were significantly perturbed at the meta-organism level. The microbiota-specific network analysis supported this observation. Networks linked to several short chain fatty acids were perturbed along with inhibited cluster of networks linked to energy cycles, such as TCA cycle and Glycolysis.
Conclusion: In conclusion, there was a significant gender-specific diversity and commensal abundance variabilities 6m TSR, which was equivalent to 18 years of human life. Functional analysis noted that the gender bias could have stemmed from the differential metabolism of lipids and some of the amino acid derivatives, such as purines. These results can inform us about the next-generation gender-/ time-/ dose-specific intervention strategies.
Disclaimer: Material has been reviewed by the Walter Reed Army Institute of Research. There is no objection to its presentation and/or publication. The opinions or assertions contained herein are the private views of the author, and are not to be construed as official, or as reflecting true views of the Department of the Army or the Department of Defense.
Research was conducted under an approved animal use protocol in an AAALAC International-accredited facility in compliance with the Animal Welfare Act and all other federal statutes and regulations relating to animals and experiments involving animals, and adheres to principles stated in the Guide for Care and Use of Laboratory Animals, NRC Publication, 2011 edition

Gut Instinct - Immune Response Evaluation: A platform for evaluation of immune response to commensal and pathogenic microbes

Michaela Welch, Jennifer Walker, Jayashree Iyer, Melissa Sprachman, Heather Jenkins, Kimberly Kelly, Alicia Meehan-Qiu, Peter Hsi, Chelsea Zhang, Lauren Hapach, Thomas Mulhern, James Cousens, Zachary Tranchemontagne, Rebecca Christianson, Elizabeth Wiellette

The microbiome and the human immune system are tightly linked, where each modulates the status and function of the other. This interdependence can be studied in the context of animal models, but studies of human microbiome:host co-regulation are hampered by the challenge of disentangling causation from correlation. Therefore, characterization of human immune and microbe interactions will benefit from ex vivo models that provide co-culture of microbes and host cells, thereby allowing controlled experiments. To meet this need, Draper is developing a novel platform that integrates key human immune cell types with intestinal epithelial cells, which provide both the barrier and the permissive interface between the host and microbiome. The ileum in the small intestine is a crucial site of exposure and a training ground for the immune system, where Peyer's patches of the epithelial lining provide a permeable interface for luminal content sampling by sentinel Dendritic cells and Macrophages. Our approach is to build a custom three-dimensional matrix similar to the lamina propria and designed to support Dendritic and T cells, in immediate contact with a relevant epithelial monolayer. This structure enables an in vitro model where commensal microbes, pathogens and other antigens can be introduced to the epithelium, and the innate and adaptive immune response can be evaluated. This presentation will summarize our combined culture of primary human epithelial cells, Dendritic cells, and T cells within the context of an electrospun matrix custom-designed to support appropriate cell interactions. In addition, preliminary experiments that integrate bacteria are underway. Future work will include development of a platform that will house the material and provide reproducible and resilient assay conditions.

Fecal DNA Extraction Methods Study

C1C Philip Golder, Junil Kim, SSgt. Arianna Baldwin, Dr. Zachary Liechty, Dr. Michael Goodson

To ensure the validity of future microbiome studies requiring the sampling and extraction of fecal DNA, it is necessary to determine how the size of fecal sample collected influences its microbial composition. In this study, we explore how variations of fecal sample collection methods influences the results of DNA extraction by varying the fecal mass collected from a sample from different individuals and observing how the mass influences microbial composition.

Considerations for using environmental microorganisms for biotechnology applications

Robyn A. Barbato

Biotechnology affords incredible opportunities to use organisms to create materials and products relevant to the military. More and more, environmental microorganisms are being considered and investigated for biotechnology and synthetic biology applications. The potential advantage to engineering an environmental isolate is its ability to survive challenging and vacillating conditions in nature. Because these microorganisms are collected from the environment, it is important to consider the ethical, legal, social, and environmental implications (ELSEI) for their use in biotechnology. This is particularly important as the number and types of environmental microorganisms for biotechnology applications grow. Here, I present ELSEI considerations for using environmental microorganisms for biotechnology with particular attention paid to land owners and commercialization of biotechnology products. I will explore ideas on how technology is ahead of the agreements and draw on my experience collecting and analyzing microorganisms from cold regions. A key takeaway is that ELSEI should be integrated early in the research plan for a readily available product.

Measurement Assurance for Innovation in Microbiome Science

Stephanie L Servetas, Monique E. Hunter, Jennifer N. Dootz, Samuel P. Forry, Jason G. Kralj, Scott A. Jackson

Appreciation for the role of microbes in our lives has been growing rapidly, but the measurement science needed to understand and fully harness microbial systems has developed at a much slower pace than the industries dependent on them demands. In all applications involving complex microbial communities, the research is hampered by the lack of standards, protocols, and technical infrastructure to allow confidence in the data and comparability. At NIST, we are developing tools to enable measurement assurance of complex microbial systems for applications in clinical diagnostics, agriculture, and the environment.

Wastewater monitoring to assess population stress levels

Taylor Jones

Wastewater-based epidemiology investigations have been used for a variety of applications including environmental issues, infectious disease, bioterrorism, and even criminal investigations. A yet unexplored use of wastewater analysis is quantifying neurotransmitters as a potential source of information to detect areas of high stress and anxiety of a given population. This study evaluated the longitudinal concentrations of the monoamines, serotonin and noradrenaline in wastewater. The composite samples were collected from five access points that addressed different population types from two different military installations. The samples were then extracted and analyzed via enzyme-linked immunosorbent assay kits and normalized to a biomarker for waste levels, Pepper Mild

Mottle Virus (PMMoV). Serotonin exhibited fluctuations over the course of the study, but not over the course of a day. Fluctuations in serotonin levels could be associated with times that would be expected to increase population stress levels, such as exams. Noradrenaline levels were at the limit of detection for the techniques utilized in this study and no conclusions could be drawn. This study has shown that wastewater is a medium to assess stress at the population level, which can provide additional information to commanders about the operational readiness of their units.

Precision Microbiome Editing with Engineered Bacteriophages

Vatsan Raman, Ph.D, Associate Professor

The human gut microbiome plays a fundamental role in health and wellness. Disruption to a healthy gut microbiome or dysbiosis is known to cause both acute and chronic illnesses. The importance of the gut microbiome to human health makes it a vulnerable target for bacterial bioweapons. Bacterial bioweapons engineered to induce dysbiosis could be delivered through food and water supplies, putting large populations at risk. The bacterial bioweapons may not be vulnerable to traditional antibiotics as the bacteria could be engineered to carry resistance cassettes. Our goal is to carry out precise, programmable edits to the gut microbiome to eliminate dysbiosis-inducing bacteria with engineered bacteriophages. We have developed a high-throughput mutational platform to engineer and deliver ~106 phage variants to target diverse host receptors that may be present in different pathogenic strains. Using this approach, we have engineered phages to eliminate two gut-linked pathogens, (a) Shiga toxin-producing Escherichia coli and (b) Salmonella, both major sources of foodborne illnesses. For each target, we can eliminate multiple strain variants, which demonstrates the adaptability of our approach to target diverse strains and to neutralize new threats as they emerge. To facilitate biocontainment, we are working toward creating phages that eliminate the target host but cannot replicate. This technology could be a powerful preventive/therapeutic approach to counter bacterial bioweapons by precision in situ microbiome editing.

Lyme-AID: Genetically Engineered Detection of Borrelia burgdorferi, the causative agent of Lyme Disease

Reginaldo Capati, Caleb Shin, Elizabeth Cassidy, Emma Redmond, Kelly Yoon, Cosmo Cao, Brandon Gadeken, Brennan Seibert, Brandon Anderson, Megan Dumond, Nathan Labarre, Ashlynn Meyers, Nathan Nelson, Jasmine Oki, Marion Posner, Amanda Schaefbauer, Melanie Grogger, Kristi McElmurry, Michael Mechikoff, David Morris, Jordan Steel, Victoria Morrison

The 2023 USAFA iGEM team's project, Lyme-AID, is a novel method to detect Lyme disease caused by the bacterium Borrelia burgdorferi2. As the most-common vector-borne disease in the United States, with no available vaccine, the risk of Lyme disease progressing to late-stage Lyme arthritis and potentially septic arthritis increases.2,4 Early detection of the disease has proven challenging due to the prolonged symptom onset of about one to two weeks.2 B. burgdorferi has been detected in interstitial fluid sampled from the skin via microneedles.5 However, the technology to test the sampled fluid remains unfeasible without laboratory equipment. The aim of this project is to create a biosensor patch equipped with microneedles to detect the spirochete in interstitial fluid and provide a visual signal during the presence of B. burgdorferi. Outer surface protein A (OspA), a protein unique to Lyme disease infection, is the primary biomarker used to detect B. borrelia, with E. coli used as the model organism expressing OspA.1,3 A three step approach (Attract, Inhibit, Detect) was used to produce immediate visual feedback during detection. Step one is to attract B. burgdorferi to the biosensor patch and also limit diffusion of the pathogen into the new host through expression of Salp12, an Ioxodes scapularis salivary gland protein that acts as a chemoattractant for B. burgdorferi. Inhibition of the pathogen will be accomplished through stimulation of the host immune system following exposure to OspA and primary antigens for B. burgdorferi which were engineered in E. coli. Finally, detection of the pathogen through an aptamer-mediated biosensor specifically designed to bind to OspA. Binding causes a conformational change in the aptamer, resulting in a reaction with gold nanoparticles and a

visual color change.

1. An antigen-targeting assay for Lyme disease: Combining aptamers and SERS to detect the OspA protein - ClinicalKey. [accessed 2023 Feb 9]. https://www.clinicalkey.com/#!/content/playContent/1-s2.0-S1549963422000144?returnurl=null&referrer=null
2. Carriveau A, Poole H, Thomas A. Lyme Disease. The Nursing Clinics of North America. 2019;54(2):261–275. doi:10.1016/j.cnur.2019.02.003
3. Hansson L, Noppa L, Nilsson AK, Strömqvist M, Bergström S. Expression of truncated and full-length forms of the Lyme disease Borrelia outer surface protein A in Escherichia coli. Protein Expression and Purification. 1995;6(1):15–24. doi:10.1006/prep.1995.1003
4. Hook SA, Hansen AP, Niesobecki SA, Meek JI, Bjork JKH, Kough EM, Peterson MS, Schiffman EK, Rutz HJ, Rowe AJ, et al. Evaluating public acceptability of a potential Lyme disease vaccine using a population-based, cross-sectional survey in high incidence areas of the United States. Vaccine. 2022;40(2):298–305. doi:10.1016/j.vaccine.2021.11.065
5. Kight E, Alfaro R, Gadila SKG, Chang S, Evans D, Embers M, Haselton F. Direct Capture and Early Detection of Lyme Disease Spirochete in Skin with a Microneedle Patch. Biosensors. 2022;12(10):819. doi:10.3390/bios12100819

Acknowledgements

We would like to thank all of those who have contributed to the planning and success of TSMC2023! We feel this long and distinguished list conveys the interest and importance of this meeting to the Department of Defense and to the microbiome field in general.
